# The Transient Receptor Potential (TRP) Channel Family in *Colletotrichum graminicola*: A Molecular and Physiological Analysis

**DOI:** 10.1371/journal.pone.0158561

**Published:** 2016-06-30

**Authors:** Mario Lange, Fabian Weihmann, Ivo Schliebner, Ralf Horbach, Holger B. Deising, Stefan G. R. Wirsel, Edgar Peiter

**Affiliations:** 1 Plant Nutrition Laboratory, Institute of Agricultural and Nutritional Sciences (IAEW), Faculty of Natural Sciences III, Martin Luther University Halle-Wittenberg, Halle (Saale), Germany; 2 Phytopathology and Plant Protection, Institute of Agricultural and Nutritional Sciences (IAEW), Faculty of Natural Sciences III, Martin Luther University Halle-Wittenberg, Halle (Saale), Germany; 3 Interdisciplinary Centre for Crop Plant Research (IZN), Martin Luther University Halle-Wittenberg, Halle (Saale), Germany; Indiana University School of Medicine, UNITED STATES

## Abstract

Calcium (Ca^2+^) is a universal second messenger in all higher organisms and centrally involved in the launch of responses to environmental stimuli. Ca^2+^ signals in the cytosol are initiated by the activation of Ca^2+^ channels in the plasma membrane and/or in endomembranes. Yeast (*Saccharomyces cerevisiae*) contains a Ca^2+^-permeable channel of the TRP family, TRPY1, which is localized in the vacuolar membrane and contributes to cytosolic free Ca^2+^ ([Ca^2+^]_cyt_) elevations, for example in response to osmotic upshock. A TRPY1 homologue in the rice blast fungus is known to be important for growth and pathogenicity. To determine the role of the TRP channel family in the maize pathogen *Colletotrichum graminicola*, proteins homologous to TRPY1 were searched. This identified not one, but four genes in the *C*. *graminicola* genome, which had putative orthologs in other fungi, and which we named *CgTRPF1* through *4*. The topology of the CgTRPF proteins resembled that of TRPY1, albeit with a variable number of transmembrane (TM) domains additional to the six-TM-domain core and a diverse arrangement of putatively Ca^2+^-binding acidic motifs. All *CgTRPF* genes were expressed in axenic culture and throughout the infection of maize. Like TRPY1, all TRPF proteins of *C*. *graminicola* were localized intracellularly, albeit three of them were found not in large vacuoles, but co-localized in vesicular structures. Deletion strains for the *CgTRPF* genes were not altered in processes thought to involve Ca^2+^ release from internal stores, i.e. spore germination, the utilization of complex carbon sources, and the generation of tip-focussed [Ca^2+^]_cyt_ spikes. Heterologous expression of *CgTRPF1* through *4* in a *tryp1*Δ yeast mutant revealed that none of the channels mediated the release of Ca^2+^ in response to osmotic upshock. Accordingly, aequorin-based [Ca^2+^]_cyt_ measurements of *C*. *graminicola* showed that in this fungus, osmotic upshock-triggered [Ca^2+^]_cyt_ elevations were generated entirely by influx of Ca^2+^ from the extracellular space. *Cgtrpf* mutants did not show pathogenicity defects in leaf infection assays. In summary, our study reveals major differences between different fungi in the contribution of TRP channels to Ca^2+^-mediated signal transduction.

## Introduction

Like any organism, fungi must perceive and respond to their environment to survive and propagate. For example, spores of plant pathogenic fungi perceive certain features of the host surface, which initiates a developmental programme that may culminate in an appressorium. This highly specialized cell allows for a pressure-mediated penetration of intact host cuticle and epidermal cell wall. This pressure, which may reach values of 5.5 MPa in *Colletotrichum graminicola*, is generated by the accumulation of osmotically active compounds [1] and needs to be sensed and tightly controlled to ensure successful penetration while preventing a bursting of the appressorium [2]. *C*. *graminicola* is a hemibiotrophic pathogen of maize, which, inside its host, passes through a short biotrophic and a longer necrotrophic phase, characterised by the controlled expression of subsets of genes [3, 4]. Again, perception of and response to the environment within the host is important for an effective colonization [5, 6]. One of the fungus' environmental parameters that may change abruptly, within a wide range, and throughout the fungal life cycle is the osmotic potential. Osmotic shock situations occur, for example, during exposure to rainwater or during the lysis of host cells.

The coupling of stimulus perception by a fungus and its responses on transcriptional or post-transcriptional levels involves numerous interacting signalling networks, including, for example, G-proteins, MAP kinases, and cyclic nucleotides [7, 8]. Calcium (Ca^2+^) is another ubiquitous second messenger in all higher organisms and plays a central role in the initiation of responses to external stimuli, including osmotic shock, and to internal cues [9, 10]. In the cytosol, Ca^2+^ binds to target proteins, such as calcineurin and calmodulin (CaM), resulting in conformational changes that modulate their activity or their interaction with other proteins. In fungi, the Ca^2+^- and CaM-activated protein phosphatase calcineurin dephosphorylates the transcription factor Crz1 allowing it to enter the nucleus and triggering transcription [11]. Deletions of either gene in filamentous fungi result in growth retardation and reduced virulence [12, 13].

Ca^2+^ signals are generated by the passive diffusion of Ca^2+^ into the cytosol, facilitated by Ca^2+^-permeable channels. The elevation of cytosolic free Ca^2+^ ([Ca^2+^]_cyt_) is terminated by the activity of Ca^2+^/H^+^ antiporters and Ca^2+^-ATPases which transport Ca^2+^ out of the cytosol [14, 15, 16]. Hence, Ca^2+^ channels are actively regulated by a signal transduction pathway, while Ca^2+^/H^+^ antiporters and Ca^2+^-ATPases respond to the increased [Ca^2+^]_cyt_. Ca^2+^-permeable channels may be activated by a number of ligands, such as inositol phosphates, cyclic nucleotides or amino acids, and by physical parameters, such as voltage or stretch of the membrane [17]. They may be either located in the plasma membrane or in membranes of intra-cellular compartments, hence mediating the entry of extracellular Ca^2+^ into the cytosol or Ca^2+^ release from internal stores, respectively. Albeit this diversity of Ca^2+^ conductances suggests a number of underlying genes, in fungi the molecular identity has been resolved for only very few channel systems. Comparative genomic analyses indicated that some fungi bear mitochondrial calcium uniporters, and some basal fungi also have genes encoding putative P2X receptors in their genomes [18, 19]. However, none of these putative fungal Ca^2+^ channel classes has been functionally analysed so far. The plasma membrane of the yeast *Saccharomyces cerevisiae* harbours a homologue of animal voltage-gated Ca^2+^ channels, Cch1, which physically interacts with another membrane protein, Mid1 [20, 21, 22, 23]. The Cch1Mid1 complex forms a high-affinity Ca^2+^ uptake system (HACS), which is activated by multiple stimuli, such as osmotic, iron, cold, and alkali stress [24, 25, 26]. Deletion of either *Cch1* or *Mid1* leads to an increased sensitivity of yeast cells to these stresses, in particular if the Ca^2+^ concentration in the medium is low. In filamentous fungi, deletion of *Cch1* or *Mid1* homologues causes reduced hyphal growth, albeit fungal species differ in their requirement of this channel system [27, 28, 29]. Next to the HACS, there also exists a low-affinity Ca^2+^ uptake system (LACS) of unclear genetic identity [22].

The vacuole represents the largest intracellular store for Ca^2+^ in yeast. The vacuolar membrane harbours a Ca^2+^-permeable channel, initially named Yeast Vacuolar Channel 1 (Yvc1), which is related to Transient Receptor Potential (TRP) channels of animals [30]. Animal TRP channels group into seven subfamilies (TRPC, TRPV, TRPA, TRPM, TRPP, TRPML, and TRPN), and most of them are permeable for Ca^2+^ [31]. Animal TRP channels locate to various endomembranes as well as to the plasma membrane. All TRP channels are supposed to contain at least six transmembrane (TM) domains and a pore loop between TM domain 5 and 6. The C- and N-termini of TRP channels are highly diverse. TRP channels are often activated in a polymodal way, i.e. a single channel integrates different stimuli, such as temperature, voltage, and ligands [31]. Fungal TRP channels form a separate subfamily [32]. In analogy to the animal TRP nomenclature, the TRP channel of yeast, Yvc1, was also denominated TRPY1. This channel is activated by cytosolic Ca^2+^ and by osmotic upshock leading to mechanical force on the vacuolar membrane [30, 32, 33, 34]. Heterologous expression of the *TRPY1* homologues of *Kluyveromyces lactis* and *Candida albicans*, as well as the filamentous plant pathogenic fungus *Fusarium graminearum* (teleomorph *Gibberella zeae*), in *S*. *cerevisiae* demonstrated a mechanosensitivity and a responsiveness to osmotic upshock, similar to TRPY1 [35, 36]. In a comparative RNAi knock-down study, Nguyen and co-workers (2008) [37] examined the importance of homologues of the yeast Ca^2+^ channels Cch1, Mid1, and TRPY1 in the rice blast fungus *Magnaporthe oryzae*. Interestingly, in this pathogen *TRPY1* was clearly more important than *Cch1* and *Mid1* for growth and virulence [37]. Similarly, hyphal growth and virulence were strongly impaired in a *trpy1* mutant of the dimorphic fungus *C*. *albicans* [38].

Despite the obvious importance of TRPY1-like channels in filamentous fungi, their functioning has been rarely analysed, with the notable exception of TRPGz from *F*. *graminearum* [36]. We therefore searched for TRPY1 homologues in the maize pathogen *C*. *graminicola*. Intriguingly, we identified four genes with similarity to *TRPY1* in this organism, which were functionally characterised by heterologous expression in yeast and sub-cellular localization. Deletion strains were analysed for [Ca^2+^]_cyt_ signal generation, germination, growth rates, tolerance to osmotic stress and Ca^2+^ starvation, as well as virulence. Surprisingly, our results differed considerably from data obtained previously on TRPY1 homologues in other fungal species, indicating that fungi vary largely in their employment of Ca^2+^ channel types.

## Materials and Methods

### Bioinformatic analyses

A tBLASTn search of the *C*. *graminicola* whole genome sequence hosted at the Broad Institute was carried out using default parameters. This identified four fragments with similarity to the TRPY1 channel (synonym Yvc1; systematic name: YOR087W) of *Saccharomyces cerevisiae*. The corresponding genes were denominated *CgTRPF1* through *CgTRPF4*. Full-length sequences of the *CgTRPF* genes were obtained by RACE-PCR as described below. Membrane topology of the CgTRPF proteins was analysed with TOPCONS (accessed at http://topcons.net/) using default settings. The putative pore loop was inferred from previous analyses [18]. Canonical Ca^2+^-binding sites were searched with PFAM (accessed at http://pfam.xfam.org/) using default settings. TRPY1 is known to contain no classical Ca^2+^-binding sites, but binds Ca^2+^ by a tetra-aspartate motif (DDDD) [34]. Therefore, motifs with four or more consecutive acidic amino acid residues (Asp and Glu) were searched in the CgTRPF protein sequences. Homologous sequences of other fungi were obtained by tBLASTn searches of their annotated genomes. Multiple sequence alignments of the full-length proteins, including 43 sequences from 20 fungal species, were performed using ClustalW [39]. Resulting alignments were trimmed with Jalview [40] (S1 File). Subsequent phylogenetic analyses were performed by neighbour joining (10,000 bootstrap replicates) using Phylip hosted at http://www.es.embnet.org/Services/. A consensus tree was created by using plottree (http://www.bioinformatics.nl/tools/plottree.html). Amino acid identity and similarity was calculated with the help of the trimmed alignments that were used for the phylogenetic analysis at http://imed.med.ucm.es/Tools/sias.html with standard settings.

### Expression analysis

Expression of *CgTRPF1* through *4* was examined by amplifying the full-length CDS from cDNA obtained from OMA-grown falcate conidia and hyphae grown on modified Leach's Complete Medium (mLCM) overlaid with a PVDF membrane [41]. Transcript abundance of *CgTRPF* genes during infection of maize was analysed by qRT-PCR and RNA-Seq. For qRT-PCR experiments, maize (cv. Mikado, KWS Saat AG, Einbeck, Germany) plants were cultivated on compost soil in a greenhouse [42]. Detached segments of the middle of the third leaf of 17-day-old plants were infected with one 10-μL drop per segment containing 10^4^ conidia in a 0.04% Tween 20 (Carl Roth) solution. Leaf segments were incubated in moist chambers at 23°C in darkness. After the indicated time, leaf discs of 8 mm diameter were excised and immediately frozen in liquid nitrogen. Four leaf discs were pooled for each point in time. Infection assays were performed in three biological replicates in consecutive weeks. Leaf discs were ground in liquid nitrogen. The resulting powder was suspended in RLT buffer (Qiagen, Venlo, The Netherlands) and processed in aliquots for RNA extraction using the PeqGold Plant RNA kit with on-column DNase I treatment according to the manual (PeqLab, Erlangen, Germany). qRT-PCR was performed using the Power SYBR Green RNA-to-C_T_ 1-step kit (Applied Biosystems—Thermo Fisher Scientific, Waltham, MA, USA). Reactions comprising volumes of 20 μL were set up according to the manufacturer’s instructions using 0.2 μM of each oligonucleotide and 50 ng RNA and executed in a MyiQ real-time detection system (Bio-Rad Laboratories, Hercules, USA). After reverse transcription at 48°C for 30 min, the resulting cDNA was denaturated for 10 min at 95°C and amplified in 50 cycles (95°C for 15 seconds, 60°C for 1 min). Calculation of the results was done according to Liu and Saint (2002) [43]. The oligonucleotides used are listed in S1 Table. As reference for normalisation, transcript levels of *HistonH3*, *Actin*, *and ILV5* were used. RNA-Seq data were obtained from the study of Schliebner and co-workers (2014) [44].

### Media and culture conditions

To induce the production of falcate conidia, the fungus was grown on oat meal agar (OMA) [45]. Colony growth assays were performed as described before [45]. Colony diameters were recorded daily for 4 to 10 days. Vegetative hyphae of *C*. *graminicola* were grown in liquid mLCM or on mLCM solidified with 1.5% agar (Carl Roth, Karlsruhe, Germany) [41]. For osmotic stress treatments, glycerol was added to mLCM agar prior to autoclaving at the indicated final concentrations. For assays testing growth on different carbon sources, mLCM, potato dextrose agar (PDA), and minimal medium supplemented with the respective carbon source were used. PDA was made of 2.4% potato dextrose broth (Formedium, Hunstanton, UK) and 1.5% agar. Minimal medium contained salt solutions as used in mLCM, 1.5% agar, and 2% of the respective carbon source (glucose, sucrose, sorbitol, mannitol, raffinose, cellulose, malate, or pectate). Sodium malate and sodium pectate were obtained by neutralizing DL-malic acid and pectic acid to pH 7.0 with NaOH, respectively. Ca^2+^-depleted SC medium was prepared as described before [45].

### RNA extraction for cloning and RACE-PCR

*C*. *graminicola* RNA was extracted from fungal mycelium grown for four days at 23°C and 30 rpm in mLCM medium, and *S*. *cerevisiae* RNA was extracted from a log-phase culture using the Spectrum Plant Total RNA Kit (Sigma). An on-column DNase I digest was performed with the RNase-free DNase I set (Omega bio-tek, Norcross, GA, USA) according to the manufacturer's instructions.

RACE-PCR was performed with the BD SMART RACE cDNA amplification kit (BD, Franklin Lakes, NJ, USA) according to the manufacturer’s recommendations. PCR products were purified from agarose gel slices using the Wizard SV Gel and PCR Clean-Up System (Promega, Madison, WI, USA). RACE-PCR products were sequenced using the BigDye Terminator v1.1 Cycle Sequencing Kit (Thermo Fisher Scientific, Waltham, MA, USA) and gene-specific oligonucleotides (S1 Table). All oligonucleotides were purchased from Eurofins MWG Operon (Ebersberg, Germany).

### Targeted gene deletion and Southern Blotting

A hygromycin B resistance cassette was amplified from pAN7-1 (GenBank No. Z32698) [46] using the oligonucleotides unihyg-F1 and unihyg-R1 (S1 Table) [47]. Deletion cassettes were generated by double-joint PCR using the oligonucleotides listed in S1 Table [48]. Transformation of the *C*. *graminicola* M2 (M1.001) wild type isolate was performed as described previously [49]. Transformants were screened by PCR for the presence of the deletion cassette. For this purpose, DNA was isolated by using a quick extraction protocol [50]. PCR-positive clones were assayed by Southern blotting for homologous integration of the deletion cassette and ectopic integration events. The probe was generated using the oligonucleotides Hph-5'-South-for and Hph-5'-South-rev (S1 Table) as described elsewhere [49]. DNA extraction and blotting were performed as described before [51].

### Yeast complementation analysis and [Ca^2+^]_cyt_ measurements

The *Saccharomyces cerevisiae trpy1*Δ deletion strain CEN.SR36-3C (Acc. No. B0257A; genotype: CEN.PK; YOR087w/088w::HIS3) was obtained from Euroscarf (Frankfurt, Germany). *trpy1*Δ cells were transformed with the plasmid pEVP11-AEQ encoding apoaequorin [52] as described elsewhere [53]. Full-length coding sequences of the four *CgTRPF* genes and the *TRPY1* gene were cloned into the *Not*I-site of the pFL61 plasmid [54]. The pEVP11-AEQ-containing *trpy1*Δ strain was transformed with these pFL61 descendants. An RT-PCR with RNA from log-phase liquid cultures was performed to confirm the presence of full-length mRNA of the *CgTRPF* genes in these strains. For luminometric analysis of [Ca^2+^]_cyt_, yeast cultures were grown on a rotating shaker in liquid SC-Leu-Ura medium containing 2 μM coelenterazine (Carl Roth) at 30°C to a final density of 1 to 5 x 10^7^ cells per mL and diluted to 1 x 10^7^ cells per mL with fresh coelenterazine-containing medium. For [Ca^2+^]_cyt_ measurements in a tube luminometer (Sirius-1, Berthold Detection Systems, Pforzheim, Germany), 20 μl of the cell suspension were used per experiment. After 1 min baseline recording, cells were treated with 200 μl of a solution (pH 7.0) containing 1.5 M NaCl, 50 mM MES, and 25 mM EGTA. Total aequorin luminescence was discharged at the end of the experiment by injecting 220 μl of a solution containing 2 M CaCl_2_ and 20% ethanol. [Ca^2+^]_cyt_ was calculated as described by Allen and co-workers (1977) [55], which normalized differences in *Aequorin* expression (i.e., total aequorin luminescence) in different strains, and which is the most commonly used equation in the mycology community. Albeit absolute [Ca^2+^]_cyt_ values obtained by this procedure may be offset from the calculated values due to the cytosolic aequorin environment, this effect should be similar in all yeast studies employing this formula.

### [Ca^2+^]_cyt_ measurements in *Colletotrichum graminicola*

To test [Ca^2+^]_cyt_ responses of *C*. *graminicola* wild type and Δ*Cgtrpf* deletion strains to osmotic upshock, the strains were transformed with the pAEQS1-G418 plasmid [45] which encodes a codon-optimized Apoaequorin [56]. Transformants were selected by using G418 (geneticin; 600 μg mL^-1^ during transformation, 150 μg mL^-1^ during selection and single-spore isolation). For [Ca^2+^]_cyt_ measurements, 1 mL mLCM agar supplemented with 10 μM coelenterazine was poured into sterile polystyrene cylinders with slip lid (36 mm diameter x 29 mm height; neoLab, Heidelberg, Germany). 300 washed macroconidia were inoculated onto the solidified medium, and the polystyrene cylinders were covered loosely to allow for gas exchange. The fungal cultures were incubated in darkness for 3 d at 23°C and 65% relative humidity. Prior to [Ca^2+^]_cyt_ measurements, cultures were overlaid with 3 mL of a solution (pH 7.0) containing 50 mM MES-KOH and 0 or 25 mM EGTA. Cylinders were covered with parafilm and incubated for 30 min in the chamber of the Sirius-1 luminometer. After 1 min baseline recording, 4 mL of a solution (pH 7.0) containing 50 mM MES-KOH and 0 or 3 M NaCl plus 0 or 25 mM EGTA were injected through the parafilm cover. Aequorin luminescence was recorded for 30 min. To discharge total aequorin at the end of the experiment, 8 mL discharge solution containing 2 M CaCl_2_ and 100 μM digitonin (Applichem, Darmstadt, Germany) were injected, and recording continued for 60 min. Data shown represent three biological repetitions performed on different days. [Ca^2+^]_cyt_ was calculated as described above.

For [Ca^2+^]_cyt_ analyses in individual hyphae, wild type and Δ*Cgtrpf* deletion strains were transformed with a pGEM-T-PtrpC-nptII-TtrpC-PtoxB-YC3.6-TtrpC plasmid [45], encoding the FRET-based ratiometric Ca^2+^ reporter protein Yellow Cameleon 3.6 (YC3.6) [57]. Measurements of tip-focussed [Ca^2+^]_cyt_ spikes during hyphal growth were performed as described before [45].

### Subcellular localization

For subcellular localization and co-localization of the CgTRPF proteins, a dual-tagging plasmid system was employed, following the cloning workflow described by Lange and co-workers (2014) [50]. Oligonucleotides used for the cloning of localization plasmids are listed in S1 Table. Transformation and microscopic analyses were performed as described before [50].

### Germination assays

Germination and appressorium formation were assayed on polystyrene and on onion epidermis. Ten-μL droplets of washed macroconidia (see above) containing 100 and 1000 spores were inoculated onto 90-mm polystyrene Petri dishes (Greiner Bio One) and the hydrophobic face of onion epidermal strips, respectively. After incubation for 24 h in a humid chamber at 23°C in darkness, infection structures were counted by phase contrast microscopy using an Axiovert 40 CFL inverted microscope (Carl Zeiss, Jena, Germany) equipped with a 10 x / 0.25 Ph1 objective for germination assays on polystyrene and an Axioskop upright microscope (Zeiss) equipped with a 20 x / 0.45 Ph 2 objective for germination assays on onion epidermis.

### Leaf segment infection assays

Segments of the third leaf of 14-day-old maize (*Zea mays* cv. Golden Jubilee) plants cultivated in an air-conditioned greenhouse were excised and incubated to assess virulence of *C*. *graminicola* as published previously [58].

## Results

### The *C*. *graminicola* genome contains four genes with similarity to the *S*. *cerevisiae* calcium channel *TRPY1*

**Fig 1:**
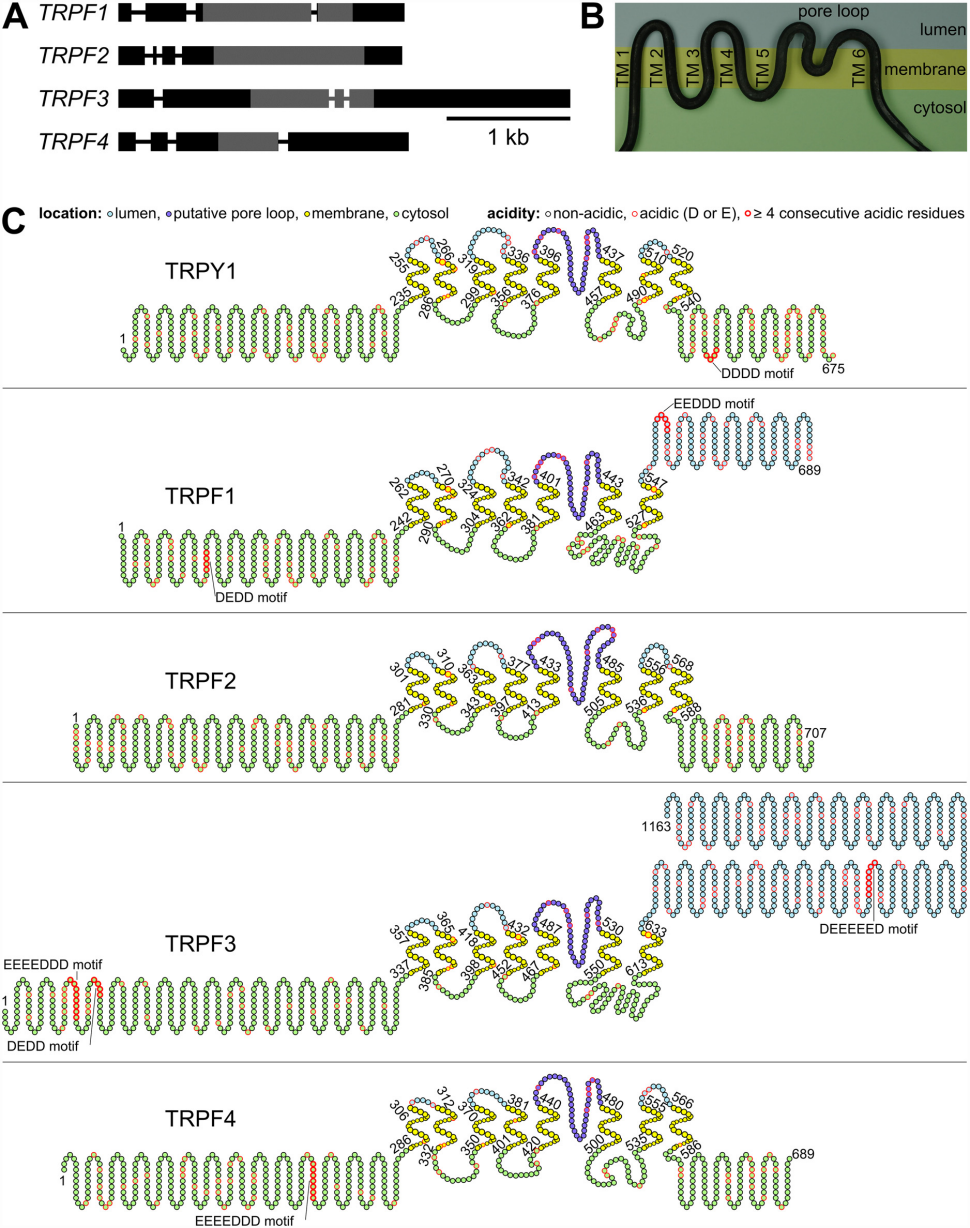
Gene structures of *C*. *graminicola TRPF* genes and predicted membrane topologies of yeast TRPY1 and *C*. *graminicola* TRPF proteins. **(A)** Gene structures of *CgTRPF* genes. Boxes: exons, lines: introns; grey: match of the initial tBLASTn search against TRPY1, black: regions identified by RACE-PCR and verified by cloning PCR. **(B)** Artistic representation (forged steel) of the TRP channel core structure containing six transmembrane (TM) domains and a pore loop between TM domain 5 and 6. **(C)** Predicted membrane topology of TRPY1 and CgTRPFs. Cytosolic amino acid residues are indicated in light green, TM domains are shown in yellow, luminal amino acid residues are depicted in light blue, and the predicted pore loop is marked in dark blue. Acidic amino acid residues [Asp (D) or Glu (E)] are indicated by a red edge, which is boldfaced in motifs of 4 or more consecutive acidic amino acid residues. One circle represents one amino acid residue. The first and the last amino acid of the whole protein, as well as of each TM domain, are enumerated.

The *C*. *graminicola* genome was searched by tBLASTn for an orthologue to TRPY1 of *S*. *cerevisiae*. Unexpectedly, not one, but four genes were identified with E-values below 10^−3^. According to the animal TRP nomenclature, we named these genes *CgTRPF1* through *4*, standing for *C*. *graminicola TRP of Fungi*. Full-length cDNA sequences were obtained by RACE-PCR and verified by cloning and RNA-Seq. In Fig 1A, the regions detected by the tBLASTn search are indicated in grey, the completed sequences in black. Comparison with the genomic regions revealed that each of the genes comprises four exons of varying length (Fig 1A). The CDSs of *CgTRPF1*, *2*, *3* and *4* consist of 2070, 2124, 3492, and 2073 bp from start to stop codon, respectively. The genomic sequences and the gene numbers can be found in S2 File. The amino acid similarities (and identities) of CgTRPF1, 2, 3 and 4 compared to TRPY1 are 60% (46%), 40% (24%), 38% (22%), and 34% (21%), respectively, based on the trimmed alignments. The gene structures that we obtained by tBLASTn searches and RACE-PCR perfectly matched the most recent genomic and transcriptomic data generated by RNA-Seq [44].

The membrane topology of yeast TRPY1 shows a Shaker-like core of six TM domains (Fig 1B), a pore loop between TM domain 5 and 6, and, in addition, two predicted additional TM domains after the sixth TM domain (Fig 1C), which were previously described as hydrophobic patches [34]. All predicted TM domains of TRPY1 and CgTRPF1 through 4 are exactly 21 amino acids long (S2 Table). There is also a high degree of conservation in the length of the luminal and cytosolic linkers of the TM domains as well as of the putative pore loop (S2 Table) [18]. CgTRPF2 and CgTRPF4 of *C*. *graminicola* have the same predicted topology as TRPY1, i.e. a six-TM-domain core and two additional TM domains. CgTRPF1 and CgTRPF3 also have the six-TM-domain core, but only one additional TM domain. Hence, the C-terminus of those proteins is predicted to reside in the lumen. In coincidence with this luminal C-terminus, CgTRPF1 and CgTRPF3 have a longer cytosolic linker between TM domain 6 and 7, as compared to TRPY1, CgTRPF2, and CgTRPF4, which have a predicted cytosolic C-terminus.

Like TRPY1, all TRPF proteins of *C*. *graminicola* have no EF-hands or other canonical Ca^2+^-binding sites detected by PFAM. However, an acidic DDDD motif in the C-terminus of TRPY1 has been shown to be important for high-affinity Ca^2+^ binding and channel activation [34]. The luminal C-terminus of CgTRPF1 and CgTRPF3 harbours acidic EEDDD and DEEEEED motifs, respectively, that may bind Ca^2+^ (Fig 1C). Additionally, acidic motifs, containing at least four consecutive amino acid residues, are present in the cytosolic N-termini of CgTRPF1, CgTRPF3, and CgTRPF4, with CgTRPF3 possessing two such stretches (Fig 1C). In CgTRPF2 there are no areas with least four consecutive acidic amino acid residues. However, the cytosolic N- and C- termini of CgTRPF2 contain many densely clustered acidic amino acid residues that may also confer an ability to bind Ca^2+^.

### The *C*. *graminicola TRPF* genes have putative orthologs in other fungi

**Fig 2:**
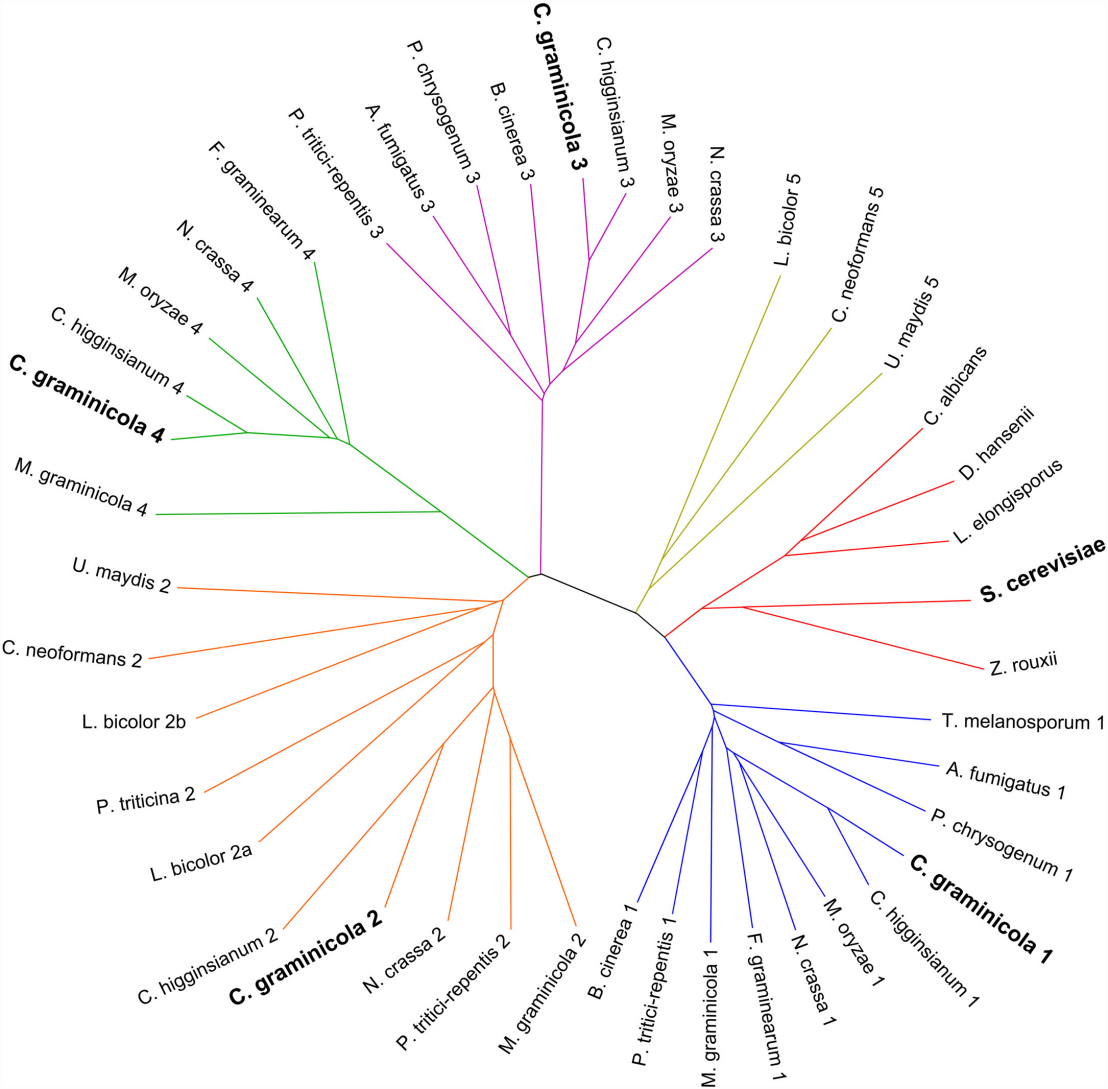
Phylogenetic tree of fungal TRP proteins. Red: yeast TRPYs; blue, orange, violet and green: TRPF groups 1, 2, 3, and 4, each containing a *C*. *graminicola* homologue; yellow: TRPF group not containing a *C*. *graminicola* homologue.

To examine whether other fungi may also possess multiple predicted proteins with similarities to TRPY1, a phylogenetically diverse set of annotated genomes of Ascomycota and Basidiomycota was searched by tBLASTn (S3 Table). The obtained sequences were aligned, trimmed, and a phylogenetic tree was generated that comprised six major branches (Fig 2). Species belonging to the Saccharomycotina harboured only one putative TRP protein and clustered on a distinct branch. *C*. *graminicola* paralogs are present in four of the five remaining branches. The fifth branch comprises only predicted proteins from Basidiomycota. In general, all examined filamentous fungi carry one to four predicted TRPF proteins. *Tuber melanosporum* contains just one TRPF which is most closely related to CgTRPF1; *Puccinia triticina* has one TRPF that co-groups with CgTRPF2. Two predicted TRPF proteins, grouping with CgTRPF1 and CgTRPF3, were found in *Aspergillus fumigatus*, *Botrytis cinerea* and *Penicillium chrysogenum*. *Fusarium graminearum* carries two TRPFs homologous to CgTRPF1 and CgTRPF4. *Cryptococcus neoformans* and *Ustilago maydis* also bear two TRPFs; one homologous to CgTRPF2, with the other one not grouping with any of the CgTRPFs. *Laccaria bicolor* has two homologues to CgTRPF2 and one not grouping with a CgTRPF. Three TRPFs were found in *Mycosphaerella graminicola* (homologous to CgTRPF1, 2, and 4), *Magnaporthe oryzae* (homologous to CgTRPF1, 3 and 4), *Pyrenophora tritici-repentis* (homologous to CgTRPF1, 2 and 3). *Colletotrichum higginsianum* and *Neurospora crassa* are the only of the analyzed species containing predicted proteins with similarity to all four CgTRPFs. There is no apparent correlation between nutritional lifestyle or pathogenicity and the number of TRPF proteins per species.

### The *C*. *graminicola TRPF* genes are expressed in axenic culture and throughout infection

**Fig 3:**
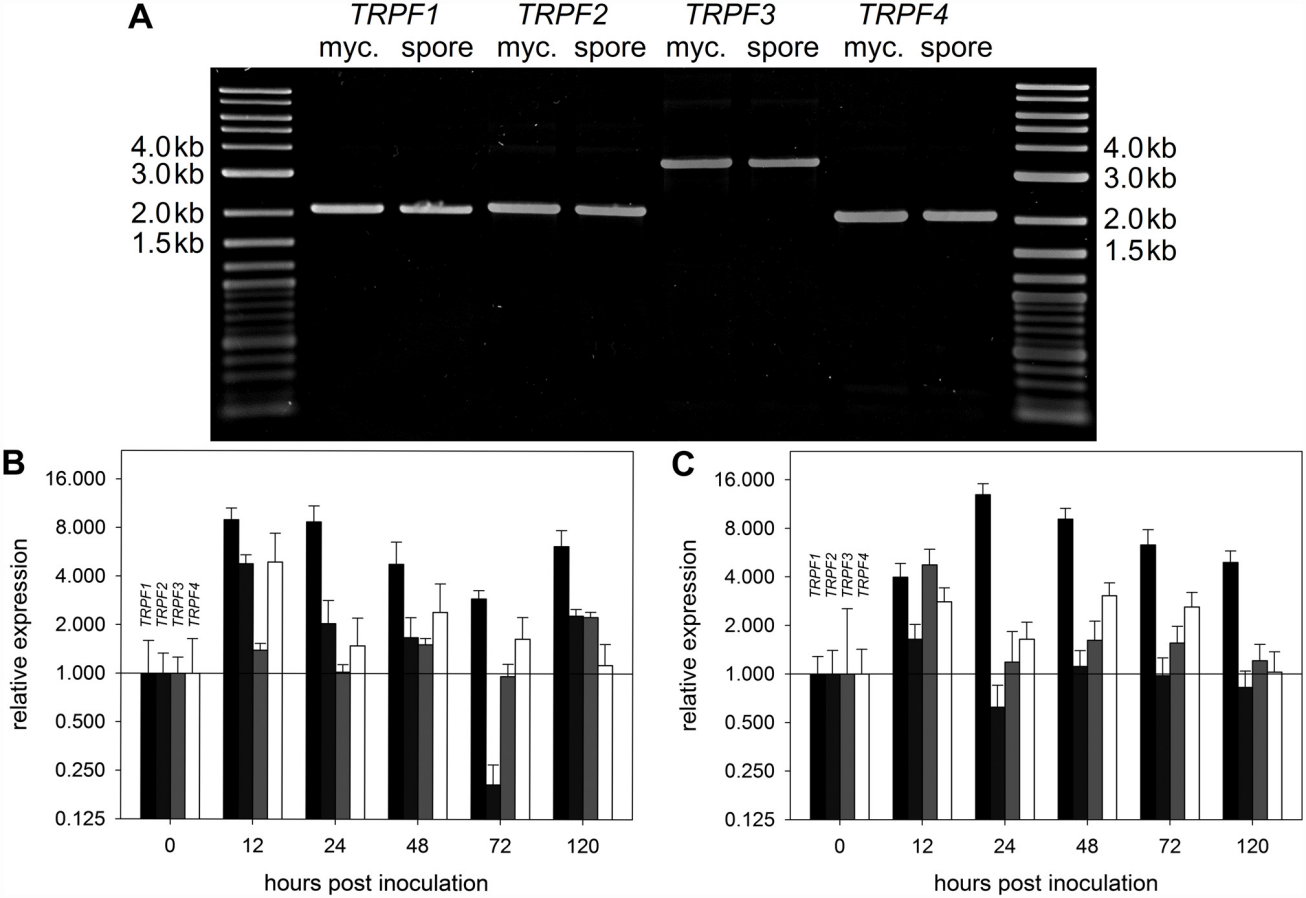
Expression profiles of the *CgTRPF* genes in axenic culture and during infection of *C*. *graminicola* on maize. **(A)** Full-length cDNA of *CgTRPF* genes was amplified from RNA extracted from *in vitro* grown mycelium and spores. Products were expected at 2098, 2152, 3522, and 2101 bp for *CgTRPF1*, *CgTRPF2*, *CgTRPF3*, and *CgTRPF4*, respectively. **(B, C)** Expression during the infection process relative to the expression in spores (0 hours post infection, hpi); black: *CgTRPF1*, dark grey: *CgTRPF2*, light grey: *CgTRPF3*, white: *CgTRPF4*. **(B)** Detached third leaves of 2.5-week-old drop-infected plants (cv. Mikado); assayed by qRT-PCR. Data are means ± SE (N = 3). **(C)** Third leaf of intact two-week-old drop-infected plants (cv. Golden Jubilee); assayed by RNA-Seq. Data are means ± SE (N = 3).

To determine whether the four *CgTRPF* genes may play a role during growth, we first determined their expression in spores and in colonies cultivated on a membrane overlying mLCM agar, as described by Lange and co-workers (2014) [41]. Full-length transcripts for all four genes were detectable in vegetative hyphae and conidial spores (Fig 3A). To determine transcript abundance throughout the infection process on maize, two independent methods were applied on two different cultivars (Mikado, Golden Jubilee). Transcript levels were assessed from 0 to 120 hours post inoculation (hpi). Irrespective of the cultivar, both, qRT-PCR and RNA-Seq experiments indicated that transcripts of all *CgTRPF* genes were clearly detectable from conidial to necrotrophic stage of infection (Fig 3B and 3C). Compared to 0 hpi, transcript levels of *CgTRPF1* were induced from 12 hpi onward by up to 13 fold in both data sets. Transcript abundances of *CgTRPF4* were also consistently elevated throughout earlier stages of the infection process, albeit to a lesser extent (Fig 3B and 3C). The transcriptomic data indicate that all four *CgTRPF* genes may play a role in all stages of growth and infection.

### The *C*. *graminicola* TRPF proteins localize at intracellular membranes

**Fig 4:**
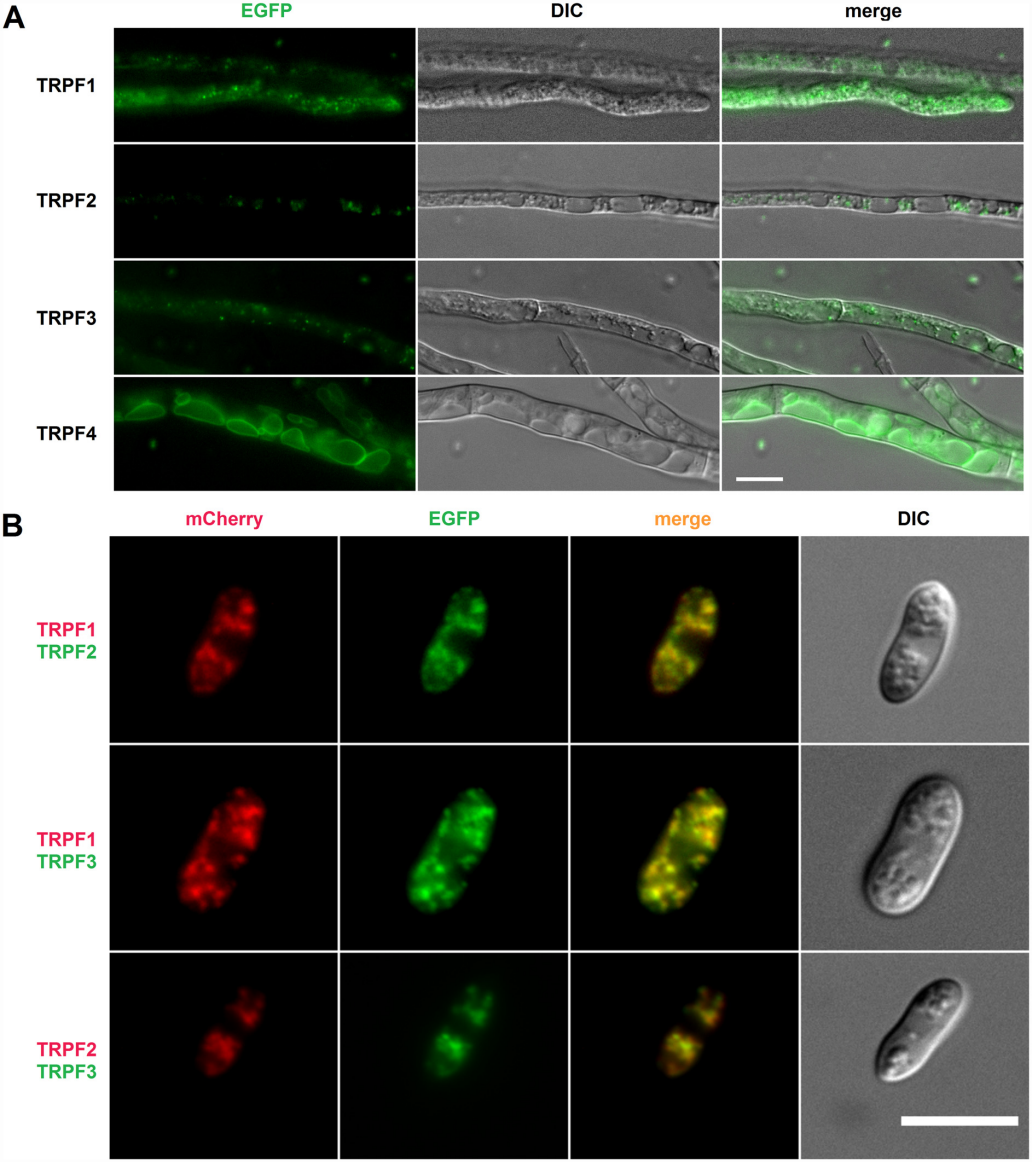
Sub-cellular localization of *Colletotrichum graminicola* TRPF proteins. **(A)** Hyphae expressing *CgTRPF1* through *4* fused to *EGFPf* driven by the respective native *CgTRPF* promoters. **(B)** Co-localisation of CgTRPF1 through 3 with each other. Oval conidia expressing *CgTRPF1* through *3* genetically fused to *mCherry* and *EGFPf* driven by the respective native *CgTRPF* promoters. Each strain was transformed with a *mCherry-*tagged *CgTRPF* gene and another *EGFPf*-tagged *CgTRPF* gene. Upper panel: *CgTRPF1-mCherry* and *CgTRPF2-EGFPf*, middle panel: *CgTRPF1-mCherry* and *CgTRPF3-EGFPf*, bottom panel: *CgTRPF2-mCherry* and *CgTRPF3-EGFPf*. Bars: 10 μm.

TRP channels of animals localize either at the plasma membrane or at membranes of intracellular organelles, while the *S*. *cerevisiae* TRPY1 channel localizes at the membrane of the central vacuole. To determine the sub-cellular localization of the CgTRPF proteins, fusions of their genes, including native promoters, to the *EGFPf* gene were created, and the *C*. *graminicola* wild type was transformed with the fusion constructs. All CgTRPF-EGFPf proteins localized at intracellular organelles (Fig 4A). However, only CgTRPF4 resided in membranes delineating large vacuoles, similar to TRPY1 in *S*. *cerevisiae*, whereas CgTRPF1, CgTRPF2, and CgTRPF3 were found in small vesicular structures. Because CgTRPF1 through 3 exhibited a similar localization pattern, we investigated whether they are present in the same compartment. To this end, the respective genes were fused to *mCherry* and combined with *EGFPf* fusion constructs in a dual-tag plasmid system [50]. The *mCherry* and *EGFPf* fusion constructs were co-expressed in *C*. *graminicola*. Fluorescence microscopy of the resulting transformants strongly suggested that CgTRPF1, CgTRPF2, and CgTRPF3 do indeed co-localize in the same cellular compartment and may thus share a common function (Fig 4B).

### Deletion strains for all four *CgTRPF* genes were obtained

As all *CgTRPF* genes were expressed in spores, plate cultures, and throughout the infection process, and since mutants of other fungi for *TRPY1* homologues show severe defects [37, 38], we analysed the role of *CgTRPF1* through *4* in growth and pathogenicity. To this end, we created deletion strains for each gene by homologous recombination. One strain for Δ*Cgtrpf1* and Δ*Cgtrpf3* and two strains for Δ*Cgtrpf2* with the desired single integration of the deletion cassette were obtained (S1 Fig). For Δ*Cgtrpf4* only strains with several integrations were obtained. For further analysis of this gene, three individual strains were chosen that showed different Southern blot patterns for one additional integration event (S1 Fig).

As there are four *TRPY1* homologs in the genome of *C*. *graminicola*, a functional redundancy of the genes is not unlikely. This may be indicated by an increased expression of the remaining *CgTRPF* genes in the deletion strains. We therefore performed qRT-PCR experiments on wild type and *Cgtrpf* deletion strains, which are shown in S2 Fig. In the deletion strains, an occasional weak upregulation of other family members was observed. However, this alteration was always well below two-fold, which does not indicate a strong compensatory response.

### Spore germination is not altered in *Cgtrpf* deletion mutants

**Fig 5:**
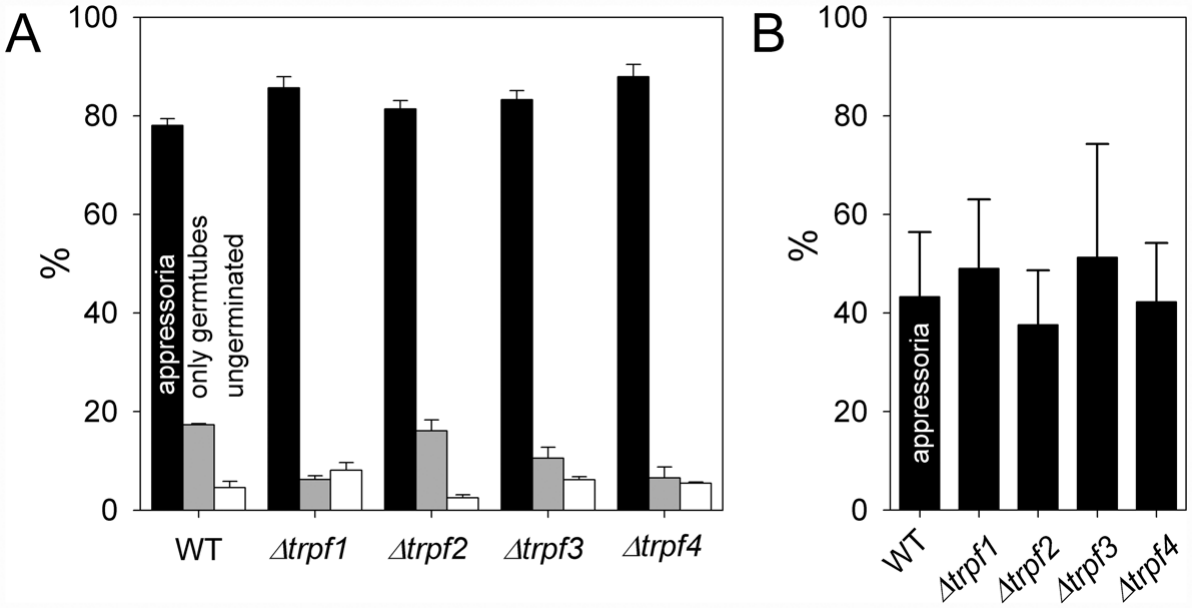
Germination of *C*. *graminicola* spores on (A) polystyrene and (B) onion epidermis. Black: germ tubes with appressoria, grey: germ tubes without appressoria, white: ungerminated conidia. On onion epidermis, only appressoria were counted. Data are means ± SE (N = 3; >100 spores per replicate).

Germination of spores and appressorium formation are initial steps in the infection process of *Colletotrichum* species, which have been reported to be dependent on Ca^2+^ release from internal stores [59]. As all *CgTRPF* genes are expressed in spores (Fig 3) and as all CgTRPF proteins are localized to endomembranes (Fig 4), a role of those proteins in spore germination appeared likely. Therefore, germination was tested on polystyrene (Fig 5A) and on onion epidermis (Fig 5B). In both types of assay, germination of the deletion strains was not reduced compared to the wild type, indicating either no role of the *CgTRPF* genes in this process or a functional redundancy of the genes.

### *Cgtrpf* deletion mutants are not defective in the utilization of complex carbon sources

**Fig 6:**
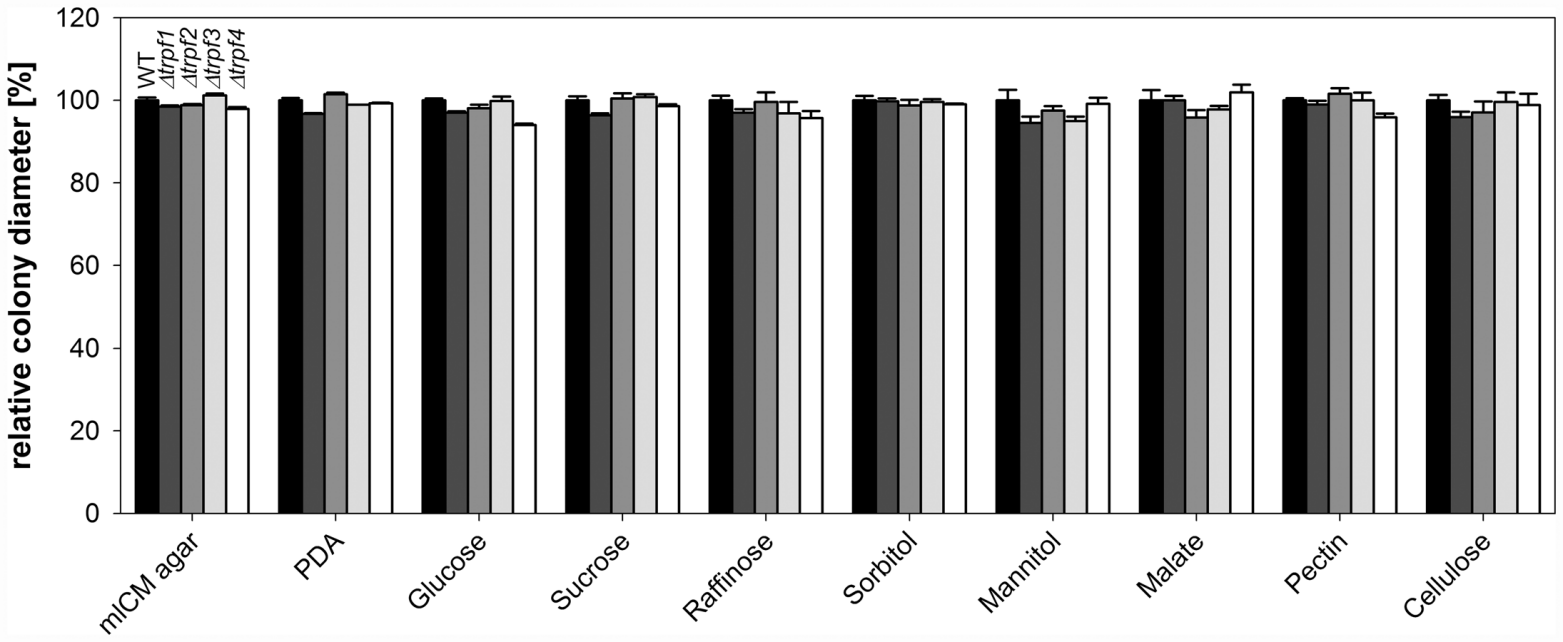
Growth of *C*. *graminicola* colonies on different carbon sources. Growth was assessed on mLCM agar, PDA, and minimal media administered with 2% of the respective carbon source. All values were normalized to the growth of the wild type on the respective medium. Colony diameter was measured 117 hours post inoculation. Data are means ± SE (N = 3).

*C*. *graminicola* is able to grow on culture media and plant tissues containing complex carbon sources. To utilize those, the fungus has to secrete hydrolytic enzymes by exocytosis, allowing the uptake of low-molecular compounds. Since exocytosis is known to be dependent on locally elevated [Ca^2+^]_cyt_, intracellular Ca^2+^ channels may have a potential impact on the secretion of enzymes that hydrolyse carbohydrates. To test whether CgTRPFs may function in this process or in the utilization of diverse carbon sources, wild type and deletion strains for all of the four genes were assayed for growth on mLCM and PDA, as well as on minimal media supplemented with glucose, sucrose, raffinose, sorbitol, mannitol, malate, pectate, or cellulose. The strains did not show any consistent and reproducible growth differences on any of the tested media (Fig 6). Hence, *CgTRPF* genes are either not required for those secretion events, or the genes are functionally redundant in this process.

### *Cgtrpf* deletion mutants are not defective in growth on low-Ca^2+^ media and in the generation of tip-focussed [Ca^2+^]_cyt_ spikes

**Fig 7:**
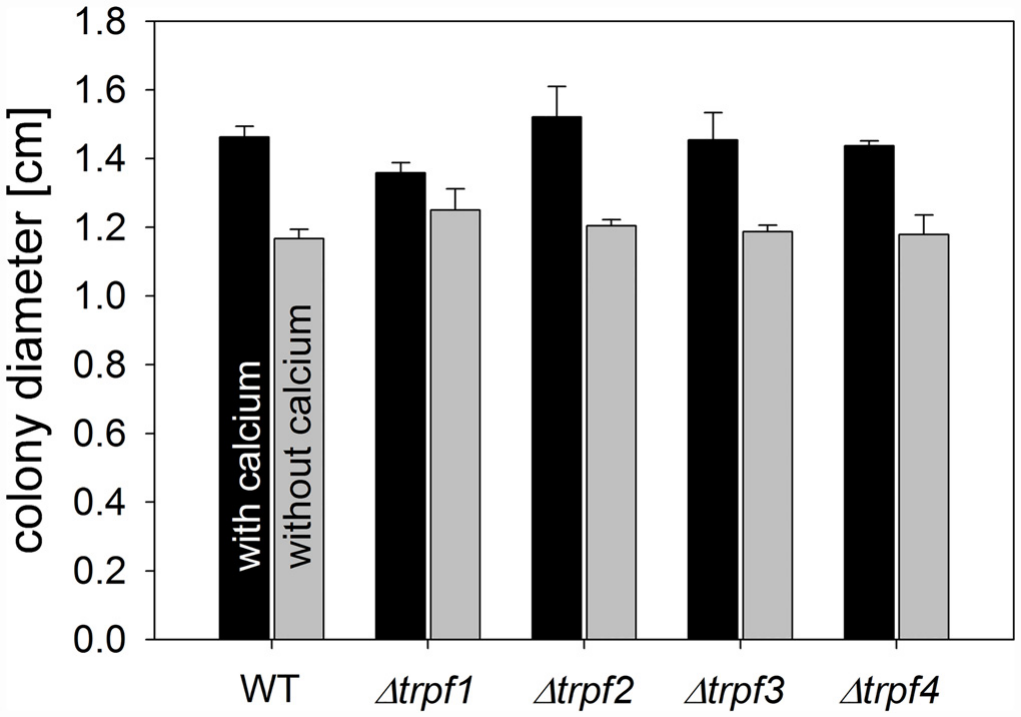
Colony growth of *C*. *graminicola* on low-Ca^2+^ media. Wild type and deletion strains were grown for 144 h on Ca^2+^-depleted SC media, and colony diameter was determined. Data are means ± SE (N = 3).

As the CgTRPF proteins have similarities to the Ca^2+^-permeable TRPY1, which is known to regulate [Ca^2+^]_cyt_ homeostasis in yeast, we tested if they play a role for growth under Ca^2+^-limited conditions. To this end, the wild type and deletion strains were cultivated on Ca^2+^-depleted SC agar medium, containing 1.7 μM total Ca^2+^, and on Ca^2+^-replete SC medium supplemented with 900 μM Ca^2+^ [45]. As previously reported [45], the wild type showed a growth reduction by around 20% on low-Ca^2+^ medium (Fig 7). This decrease in growth was also apparent, but not exacerbated, in the *Cgtrpf* mutant strains (Fig 7). Therefore, a role of CgTRPF proteins during Ca^2+^ starvation is unlikely.

**Fig 8:**
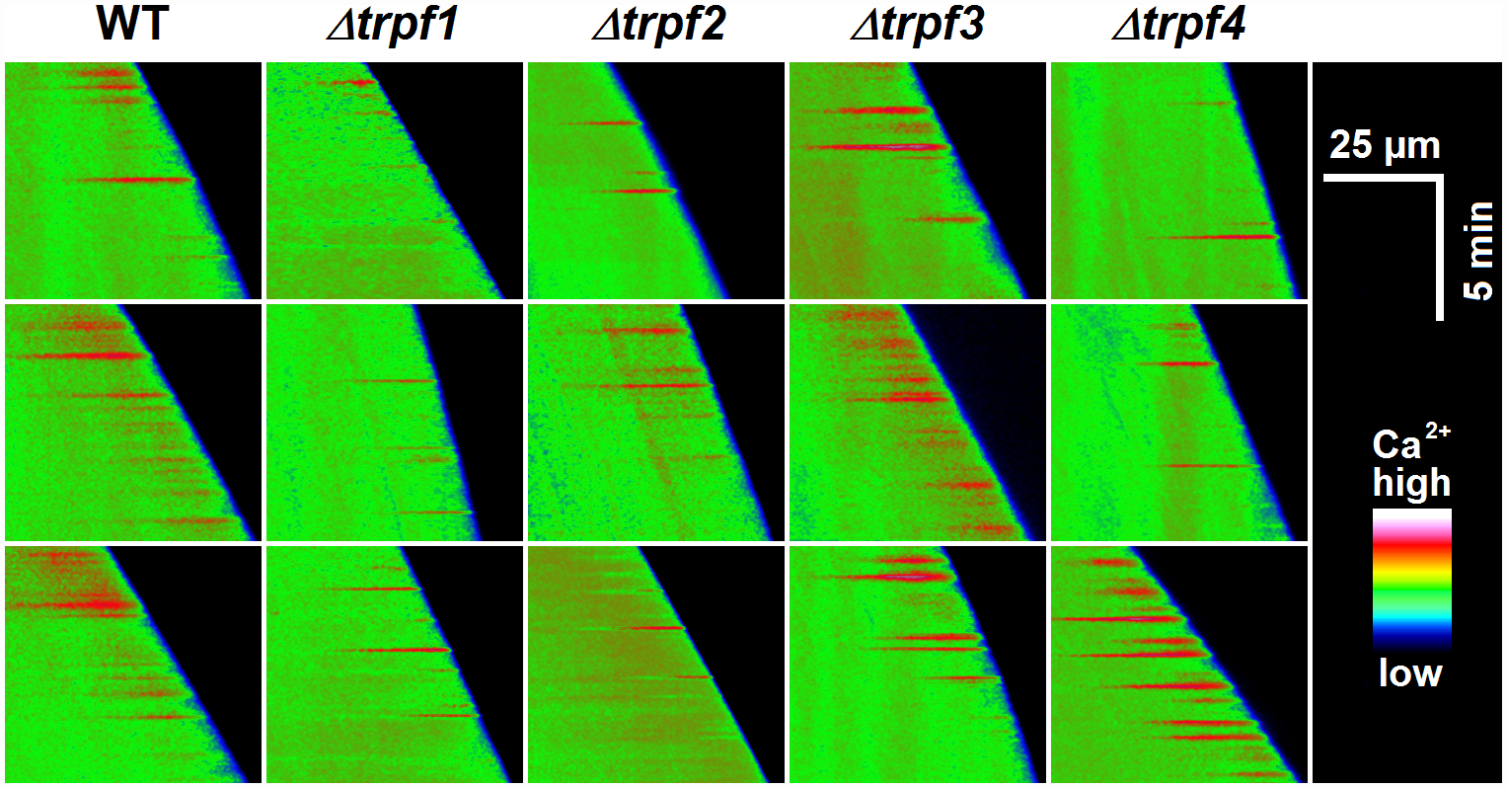
Yellow Cameleon-based measurements of [Ca^2+^]_cyt_ in individual hyphae. Kymographs of individual hyphae of wild type and deletion mutants. Each horizontal pixel line represents the mean [Ca^2+^]_cyt_ of a 5-pixel-wide ROI in the middle of each hypha. ROIs of subsequent images, acquired every 2 sec, were plotted one below the other. The slope of the kymographs, read from top to bottom, thus indicates the growth rate. Relative [Ca^2+^]_cyt_ is displayed in false-colour using the RGB rainbow scale. All measurements were performed on hyphae growing at an mLCM agar-glass interface [45].

To directly analyse a possible role of the CgTRPF proteins in the generation of [Ca^2+^]_cyt_ signals, wild type and *Cgtrpf* deletion mutants were transformed with the Ca^2+^ reporter Yellow Cameleon 3.6, and individual hyphae were examined by ratiometric fluorescence microscopy. As reported previously [45], the *C*. *graminicola* wild type showed tip-focused spikes of high [Ca^2+^]_cyt_ during hyphal growth in a highly variable manner (Fig 8). It has been suggested that in *N*. *crassa*, a tip-focussed [Ca^2+^]_cyt_ gradient is generated by Ca^2+^ release from intracellular vesicles [60]. We therefore tested whether any of the CgTRPFs may contribute to the apical [Ca^2+^]_cyt_ spikes of *C*. *graminicola*. However, all mutants still showed [Ca^2+^]_cyt_ spiking at the hyphal tip (Fig 8), with the spike occurrence being highly variable between individual hyphae.

To observe [Ca^2+^]_cyt_ spike occurrence in undisturbed whole colonies, we developed a protocol based on aequorin luminometry [45]. Similar to our previous study [45], the wild type generated distinct [Ca^2+^]_cyt_ spikes during growth, which had a similar duration as the spikes observed in individual tips (not shown). The rate of spike occurrence was not reduced in any of the deletion strains (S3 Fig).

### CgTRPF proteins do not function as osmotic stress sensors

**Fig 9:**
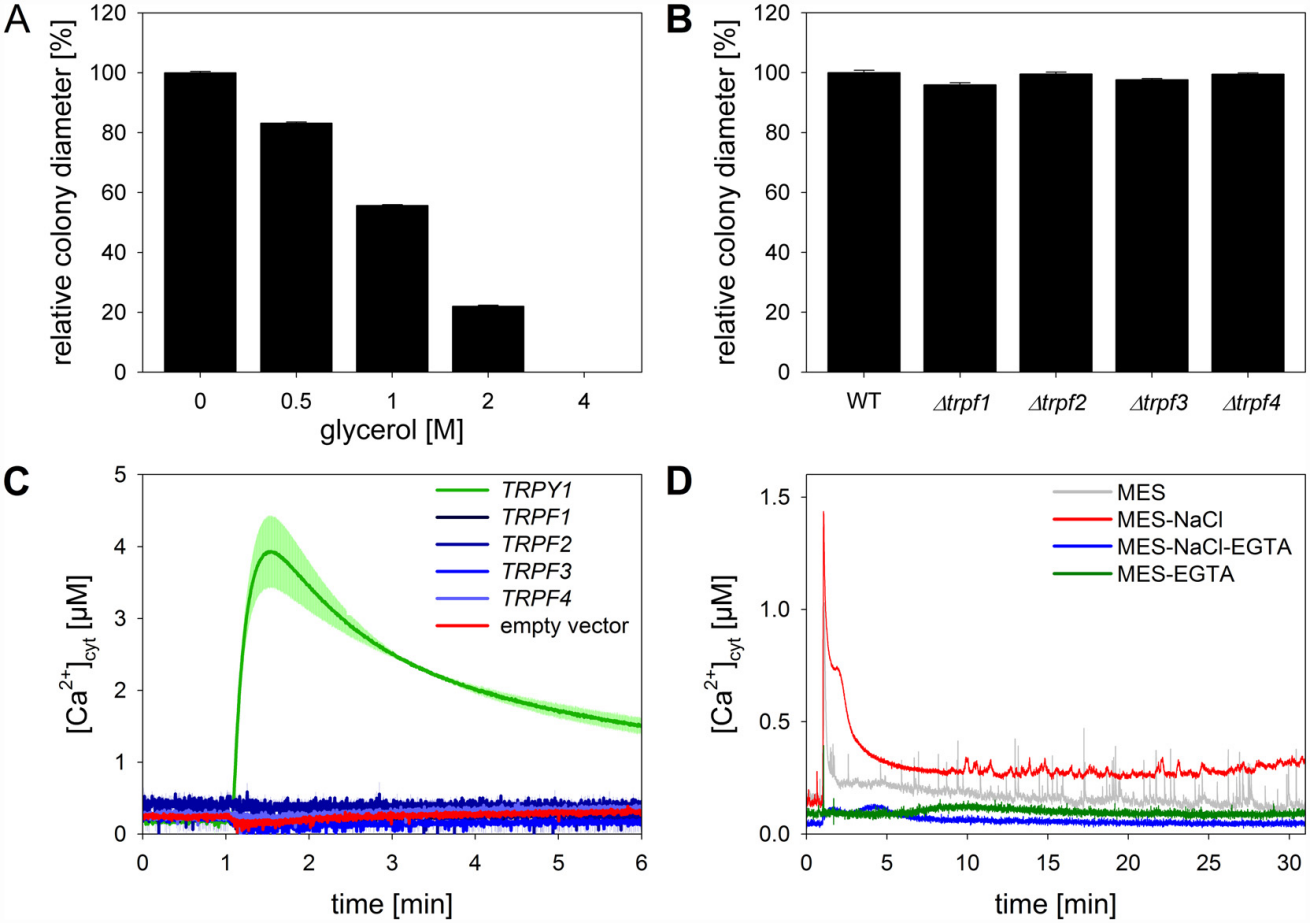
Response of mycelial growth of *C*. *graminicola* and [Ca^2+^]_cyt_ of *C*. *graminicola* and yeast to hyperosmotic stress. **(A, B)** Mycelial growth of *C*. *graminicola* osmotically stressed with glycerol. **(A)** Colony diameter of wild type stressed with varying concentrations of glycerol, normalized to unstressed colonies. **(B)** Wild type and deletion strains stressed with 0.5 M glycerol, normalized to the wild type. Colony diameters were determined 122 hours post inoculation. Data are means ± SE (N = 3). **(C, D)** [Ca^2+^]_cyt_ response of yeast and *C*. *graminicola* to NaCl measured by aequorin luminescence. **(C)** Response of *trpy1*Δ yeast mutant cells transformed with the indicated vectors to a solution (pH 7.0) containing 1.5 M NaCl, 50 mM MES-KOH, and 25 mM EGTA. Treatment was started at 1 min. Red line: empty pFL61 vector (negative control); green line: pFL61-ScTRPY1 (positive control); blue lines: yeast strains transformed with pFL61 containing *CgTRPF1* through *4*. Data are means ± SE (N = 3). **(D)** [Ca^2+^]_cyt_ measurements on *C*. *graminicola* wild type colonies. Whole colonies were pre-treated with 50 mM MES-KOH (pH 7.0) for 30 min prior to recording, followed by treatment with a solution (pH 7.0) containing 50 mM MES-KOH and no NaCl (grey line) or 1.5 M NaCl (final concentration; red line). To abolish the influx of extracellular Ca^2+^, colonies were pre-treated with a solution (pH 7.0) containing 50 mM MES-KOH and 25 mM EGTA for 30 min prior to measurement, followed by treatment with a solution (pH 7.0) containing 50 mM MES-KOH, 25 mM EGTA, and no NaCl (green line) or 1.5 M NaCl (final concentration) (blue line). Treatment solutions were added after 1 min of recording. Traces show individual measurements in order to demonstrate [Ca^2+^]_cyt_ spikes in the MES-KOH control treatment. Replicate measurements can be found in S4 Fig.

TRPY1 is known to act as a sensor for osmotic disturbance in *S*. *cerevisiae*. To analyse whether CgTRPFs of *C*. *graminicola* may share this function, deletion mutants were analysed for growth under osmotic stress conditions. As expected, growth of the *C*. *graminicola* wild type strain on mLCM agar was increasingly inhibited by increasing glycerol concentrations (Fig 9A). All deletion mutants of the four *CgTRPF* genes were inhibited similar to the wild type on medium containing 0.5 M glycerol (Fig 9B).

A possible redundancy of the CgTRPF proteins in osmotic sensing was investigated by heterologous expression in yeast harbouring the [Ca^2+^]_cyt_ reporter apoaequorin. *S*. *cerevisiae* responds to hyperosmotic stress with a Ca^2+^ influx from the extracellular medium and a concomitant release of Ca^2+^ from the vacuole mediated by TRPY1 [32]. Chelation of extracellular Ca^2+^ renders the osmotic upshock-triggered [Ca^2+^]_cyt_ elevation absolutely dependent on TRPY1. Under those conditions, a *trpy1*Δ deletion mutant carrying the empty vector pFL61 did not show any [Ca^2+^]_cyt_ response to an osmotic upshock exerted by 1.5 M NaCl (Fig 9C, red line). As expected, the transient increase in [Ca^2+^]_cyt_ after application of osmotic stress was restored in transformants complemented with the native *TRPY1* from *S*. *cerevisiae* (Fig 9C, green line). To test if any of the *C*. *graminicola* TRPF proteins mediates a Ca^2+^ flux in response to osmotic upshock, the full-length cDNAs of *CgTRPF1* though *4* were constitutively expressed in the *trpy1*Δ mutant. Expression of the *CgTRPF* genes was confirmed by RT-PCR (S5 Fig). Surprisingly, none of the *CgTRPF* genes complemented the [Ca^2+^]_cyt_ response of the yeast mutant (Fig 9C, blue lines), suggesting that they may either not be sensitive to osmotic shock or not active, e.g. due to problems with heterologous expression.

Since the *CgTRPF* genes did not complement the *trpy1*Δ yeast strain, we asked whether this fungus, like *S*. *cerevisiae*, responds to hyperosmotic shock with a [Ca^2+^]_cyt_ transient that is partially generated by Ca^2+^ release from internal stores. To this end, we employed the apoaequorin-harbouring wild type strain. Treatment of whole colonies with buffer alone caused a short and small response that phased out entirely after about 3 min (Fig 9D, grey line). This baseline was stable until the end of the measurement at 30 min except for very short [Ca^2+^]_cyt_ spikes in the range of a few seconds, as described above (S3 Fig). Treatment with a solution containing 1.5 M NaCl evoked a large initial peak in [Ca^2+^]_cyt_ that was followed by a shoulder and a sustained elevation at a level well above the baseline (Fig 9D, red line). This strong response was nearly completely prevented by the addition of EGTA, which chelates extracellular Ca^2+^ (Fig 9D, blue line). This indicates that, in contrast to *S*. *cerevisiae*, the hyperosmotic stress-triggered [Ca^2+^]_cyt_ response of *C*. *graminicola* is sourced nearly entirely from the external medium. EGTA alone provoked no discernible response (Fig 9D, green line).

### *Cgtrpf* deletion mutants are unaffected in pathogenicity

**Fig 10:**
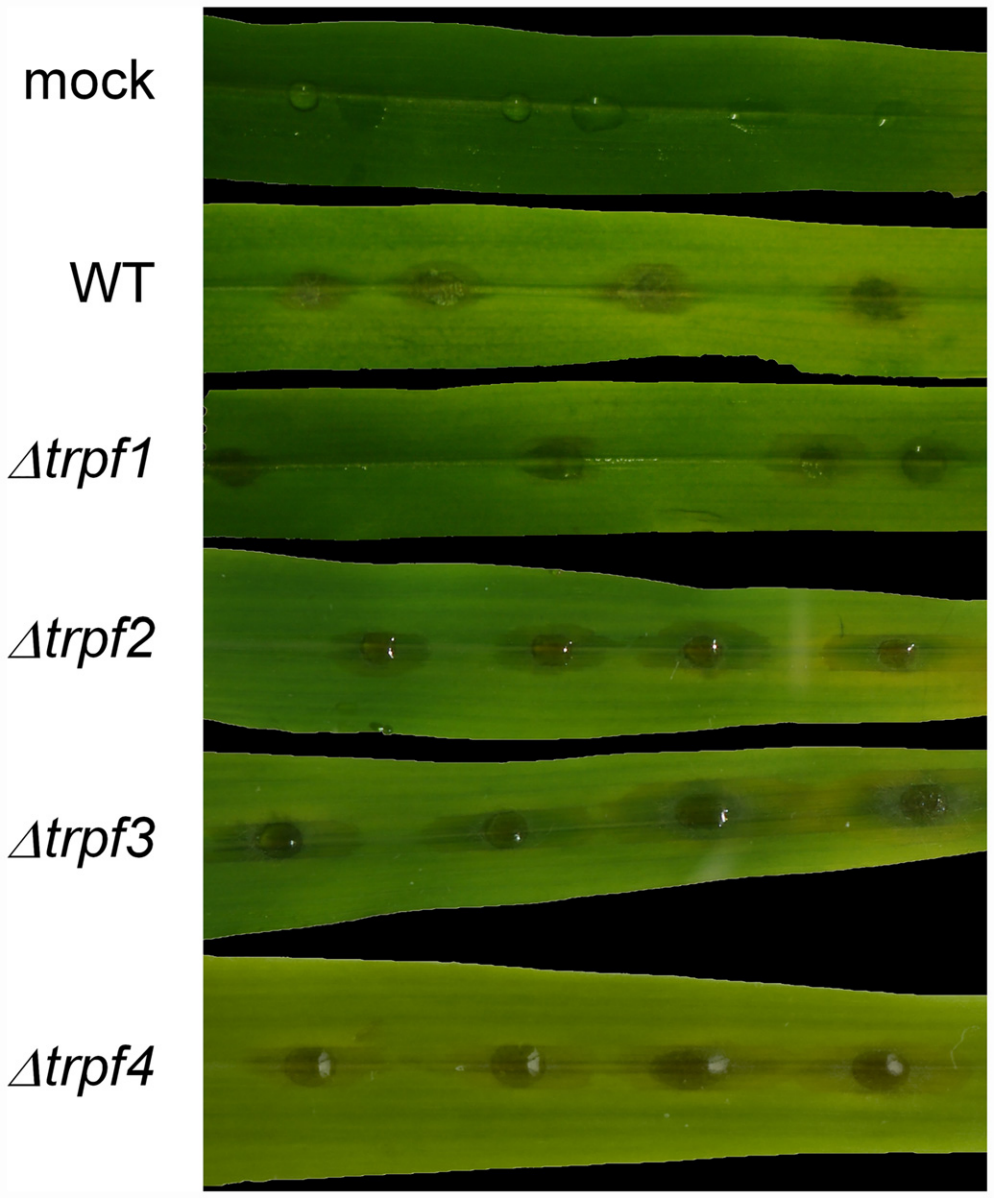
Detached-leaf infection assay. Sections of the third leaf of two-week-old maize plants (cv. Golden Jubilee) were infected with 10^4^ spores of *C*. *graminicola* wild type or *Cgtrpf1* through *4* deletion mutants per 10-μL drop, or mock-infected with 10 μL of 0.02% Tween 20 in bidistilled water. Representative images of three biological replicates are shown; the experiment was repeated twice with similar results.

As all assays failed to identify a possible involvement of *C*. *graminicola* TRPF proteins in various aspects of growth and environmental responses, and as all *CgTRPF* genes were expressed during infection of maize (Fig 3), we tested the deletion strains for virulence, which integrates a large array of sensing and signalling mechanisms. In a detached leaf segment assay, none of the mutants exhibited symptoms that differed noticeably from those of the wild type (Fig 10).

## Discussion

A number of pharmacological studies have suggested a role of Ca^2+^ release from internal organelles in the regulation of various developmental processes in fungi, such as germination [59] and hyphal tip elongation [60], as well as in responses of fungi to environmental stimuli, such as changes in osmolarity [24, 32, 56]. In *S*. *cerevisiae*, the Transient Receptor Potential channel homologue TRPY1 is a Ca^2+^-permeable channel in endomembranes that has been demonstrated to contribute to the generation of [Ca^2+^]_cyt_ signals [30]. In support of a pivotal role of this class of ion channels in fungi, the knock-down of a *TRPY1* homologue in *M*. *oryzae* resulted in a drastically reduced colony growth [37]. We therefore considered this family as a potential target for plant protection strategies against the devastating maize pathogen *C*. *graminicola*, and analysed its involvement in growth, environmental responses, and pathogenicity of this fungus. Our initial database queries revealed that this gene family was expanded in most filamentous fungi, with up to four members in the examined genomes, while the genomes of all examined yeasts contain only a single *TRPY1* homologue. This is in good agreement with a comparative genomic analysis pointing to a possible expansion of the *TRP* family in filamentous fungi [18]. The members in filamentous fungi, which we denominate *TRPF*s, cluster in five subgroups. In the genome of *C*. *graminicola*, four *TRPF* genes were found. All were expressed throughout development and infection, and all encoded proteins were localized in intracellular organelles. Further on, the proteins display a membrane topology similar to the yeast TRPY1 channel, albeit with a variable number of TM domains additional to the core of six TM domains. CgTRPF1, 3, and 4 also have acidic motifs that are similar to the Ca^2+^-binding tetra-aspartate motif of TRPY1 [34]. These motifs are predicted to reside in cytosolic or luminal termini. Interestingly, a luminal Ca^2+^-binding site and regulation of channel activity by luminal Ca^2+^ have been demonstrated for the Two Pore Channel 1 (TPC1) [61], a vacuolar cation channel in plants [62]. CgTRPF1 and CgTRPF3 may thus sense and be regulated by luminal [Ca^2+^]. Collectively, an important role of these genes in Ca^2+^-related processes, in particular those involving Ca^2+^ release from internal stores, seemed likely, but could not be unveiled in our experiments.

### *C*. *graminicola* TRPFs are not activated by osmotic upshock

In *S*. *cerevisiae*, the only TRP member, TRPY1 (Yvc1), forms a stretch-activated Ca^2+^-permeable cation channel in the vacuolar membrane [30, 33] that contributes to the generation of the hyperosmotic-shock-triggered [Ca^2+^]_cyt_ signal [32]. By complementation analysis of the *trpy1*Δ mutant, mechanosensitivity and osmotic response were also shown for the TRPY1 homologues of the yeasts *K*. *lactis* and *C*. *albicans* [35], as well as of the filamentous fungus *Fusarium graminearum* [36]. In contrast, none of the *CgTRPF* genes from *C*. *graminicola* complemented the defective [Ca^2+^]_cyt_ response of the *S*. *cerevisiae trpy1*Δ mutant to osmotic upshock. This might indicate that technical problems of the heterologous expression system (e.g. incorrect folding, missing interaction partners, insufficient stabilization against protein degradation, or mislocalization of the CgTRPF proteins) have prevented yeast complementation. However, we consider this as not very likely because other TRP channels are functional in this system. Alternatively, the failure to complement the *trpy1*Δ response to osmotic upshock may indicate that not all TRPF proteins act in the perception of osmotic stress. To further test this presumption, the response of *C*. *graminicola* to hyperosmotic shock was analysed. In *C*. *graminicola*, 1.5 M NaCl triggered a massive Ca^2+^ influx via the plasma membrane, but, unlike in yeast, a negligible release of Ca^2+^ from internal stores. Since TRPF channels of *C*. *graminicola* were localized to intracellular membranes, these results further substantiate the idea that in *C*. *graminicola* TRP proteins are not involved in Ca^2+^ release into the cytosol upon hyperosmotic stress.

### *C*. *graminicola* TRPFs are dispensable for hyphal growth in axenic culture

A further process that we considered likely to be regulated by TRPF proteins is the utilization of complex carbon sources, which relies on the exocytosis of hydrolytic enzymes. In yeast, membrane fusion, a prerequisite of exocytosis, is dependent on the activation of calmodulin by Ca^2+^ release from the fusing vesicles [63]. Furthermore, tricalbins, which contain three Ca^2+^-binding C2 domains, and which are homologous to Ca^2+^-activated synaptotagmin in animals, have been linked to membrane fusion in yeast [64]. However, despite the presumed [Ca^2+^]_cyt_ dependence of enzyme secretion, the *Cgtrpf* deletion strains did not differ from the wild type in their growth on complex carbon sources.

Growth of *C*. *graminicola* colonies is very sensitive to the inhibitors 2-APB and capsazepine, which block a range of TRP channels in animals [45]. However, none of the *Cgtrpf* deletion stains showed a diminished growth potential on various standard media, or on media with complex carbon sources, all containing high amounts of Ca^2+^. Since release of Ca^2+^ from intracellular stores might become important for growth under Ca^2+^-limiting conditions, we cultivated the strains on Ca^2+^-depleted medium, which causes a moderate growth depression in the wild type. However, growth depression in *Cgtrpf* deletion strains was not more severe than in the wild type. Hence, individual CgTRPF proteins are dispensable for growth in Ca^2+^-limiting environments.

During undisturbed growth, filamentous fungi, including *C*. *graminicola*, generate short tip-focussed [Ca^2+^]_cyt_ pulses that can be visualized by fluorescence ratio imaging microscopy of the Yellow Cameleon (YC) reporter protein [45, 65, 66]. Unlike in other tip-growing systems, such as pollen tubes [67], these [Ca^2+^]_cyt_ pulses are apparently not related to growth kinetics, but may rather be involved in environmental sensing [45]. [Ca^2+^]_cyt_ spikes of short duration are also detectable on whole-colony level as luminescence of the Ca^2+^ reporter aequorin [45]. In *N*. *crassa*, a tip-focussed Ca^2+^ gradient has been suggested to be maintained by Ca^2+^ release from intracellular vesicles [60]. However, in *Cgtrpf* deletion mutants, [Ca^2+^]_cyt_ spike generation was affected neither on single-hypha level nor on whole-colony level. This corresponds well to the fact that on whole-colony level, spike occurrence was nearly completely abolished by chelation of extracellular Ca^2+^ [45], suggesting that those [Ca^2+^]_cyt_ spikes depend on Ca^2+^ influx rather than Ca^2+^ release from internal stores.

### Deletion of *C*. *graminicola TRPF*s does not impede pathogenicity

The *CgTRPF* genes were expressed throughout the infection process of *C*. *graminicola* on maize plants. Interestingly, there was a transcriptional regulation of the genes by up to 13-fold during the course of infection. This is unexpected for ion channels, which are primarily regulated on post-translational level. However, regulation of TRP channels on mRNA level is also known for a number of mammalian family members, such as *TRPC1*, *TRPC3*, *TRPV4*, and *TRPV6* [68, 69, 70].

Leaf segment infection assays were performed to integrate all pathogenic processes from spore germination to leaf necrosis [71]. These assays did not indicate pathogenicity defects in any of the *Cgtrpf* deletion strains. This is in stark contrast to the phenotype of a *trpf1* RNAi knockdown mutant of *M*. *oryzae*, which showed a severely repressed virulence [37]. However, the *M*. *oryzae* genome bears only two *TRPF* genes, so that in *C*. *graminicola*, there may be higher degree of functional redundancy between the family members, albeit their topologies and structures vary. To resolve this question, we attempted a quadruple-RNAi knockdown approach, employing a vector system that has previously been successfully used to diminish expression of a *C*. *graminicola* β-1,3-Glucan Synthase-encoding gene [5]. Unfortunately, RNAi-based knockdown of *CgTRPF* genes was not very efficient, with their expression being decreased by not more than 50% (data not shown). Quadruple-RNAi strains did not show any phenotypic differences to the wild type during the strain selection process and were still pathogenic in leaf segment assays (data not shown). Unfortunately, it was therefore not possible to analyse a functional redundancy of the four *CgTRPF* genes, which might be the cause of the absence of phenotypical alterations in any of the single *Cgtrpf* mutants. Albeit a redundancy was not obvious on transcriptional level in the deletion strains, a redundancy may also occur on the level of protein activity. It is also possible that the *TRPF* genes of *C*. *graminicola* are important under conditions not tested in this study, but occurring in its native habitat, such as extreme temperatures, high light intensities, or interaction with other microorganisms.

### Evidence for a functional diversification of orthologous Ca^2+^ channels in fungi

In contrast to *M*. *oryzae*, TRPF proteins in *C*. *graminicola* are not essential for colony growth and virulence. Furthermore, these channels do not mediate a [Ca^2+^]_cyt_ elevation after osmotic upshock, as shown for homologues from other fungal species. This indicates a functional diversification of this ion channel family in fungi. A change in functionality can be similarly observed in an animal TRP family member, TRPV1, the receptor for capsaicin (the spicy component of hot chilli pepper): Rabbits are about 100 times less sensitive to capsaicin than humans and rats, and birds are completely insensitive. A molecular basis for these drastic differences lies in just two point mutations in the *TRPV1* gene [31]. Hence, even small variations may also render the TRPF orthologs functionally highly diverse in different fungal species.

Species-specific differences in the regulation of fungal growth have also become apparent for the Cch1/Mid1 complex that mediates Ca^2+^ uptake across the plasma membrane. In *Aspergillus nidulans* a deletion of *Cch1* and/or *Mid1* results in drastically reduced colony growth even on complete media [27], whereas in *Cryptococcus neoformans* and *Botrytis cinerea* the deletion of *Cch1* and/or *Mid1* affects growth only under severe Ca^2+^ limitation, and deletion strains of *B*. *cinera* are phenotypically indifferent from the wild type inside their native host [28, 29]. These examples support the notion that components of the Ca^2+^ signalling toolbox may be employed differently in different fungi and that results may not always be simply extrapolated from one species to another.

## Supporting Information

S1 Fig Genomic Southern Blots of the deletion mutants for *CgTRPF1* through *4*. Genomic DNA was digested using the indicated restriction endonucleases and probed with a digoxigenin-labelled probe binding to the 5’ region of the *hygromycinB phosphotransferase* gene. Clones used in this study are indicated in red.
(TIF)

S2 Fig Relative expression of the *CgTRPF* genes in *C*. *graminicola* wild type and in strains deleted for individual *CgTRPF* genes. Strains were cultivated for 3.5 days on mLCM agar using the PAAP protocol [41] and assayed by qRT-PCR. Black: *CgTRPF1*, dark grey: *CgTRPF2*, middle grey: *CgTRPF3*, light grey: *CgTRPF4*. Data are means ± SE (N = 3).
(TIF)

S3 Fig Relative [Ca^2+^]_cyt_ spiking rate during colony growth of *C*. *graminicola*. Colonies of *C*. *graminicola* wild type and *Cgtrpf1* through *4* deletion strains expressing apoaequorin were grown for 80 h in 35-mm Petri dishes on mLCM agar supplemented with 10 μM coelenterazine. [Ca^2+^]_cyt_-dependent luminescence was detected for 20 min. Data are the means ± SE (N = 4).
(TIF)

S4 Fig Additional repeats of the aequorin luminescence measurements of [Ca^2+^]_cyt_ responses of *C*. *graminicola* to NaCl. Whole colonies were pre-treated with 50 mM MES-KOH (pH 7.0) for 30 min prior to recording, followed by treatment with a solution (pH 7.0) containing 50 mM MES-KOH and no NaCl (grey line) or 1.5 M NaCl (final concentration; red line). To abolish the influx of extracellular Ca^2+^, colonies were pre-treated with a solution (pH 7.0) containing 50 mM MES-KOH and 25 mM EGTA for 30 min prior to measurement, followed by treatment with a solution (pH 7.0) containing 50 mM MES-KOH, 25 mM EGTA, and no NaCl (green line) or 1.5 M NaCl (final concentration; blue line). Treatment solutions were added after 1 min of measurement. Traces show single measurements in order to demonstrate [Ca^2+^]_cyt_ spikes in the MES-KOH control treatment.
(TIF)

S5 Fig Expression of *CgTRPF* genes in transformed *S*. *cerevisiae trpy1*Δ strains. Full-length cDNAs of the *TRPF* genes were amplified from RNA extracted from log-phase cultures of *S*. *cerevisiae trpy1*Δ transformed with pFL61-CgTRPF1 through pFL61-CgTRPF4. Products were expected at 2098, 2152, 3522, and 2101 bp for *CgTRPF1*, *CgTRPF2*, *CgTRPF3*, and *CgTRPF4*, respectively. RT: + reverse transcriptase added in cDNA synthesis, − reverse transcriptase omitted in cDNA synthesis.
(TIF)

S1 File Tree alignments. (TXT)

S2 File *C*. *graminicola TRPF* gene sequences and gene numbers. Exons are indicated in uppercase letters, introns are indicated in lowercase letters.
(TXT)

S3 File *C*. *graminicola* TRPF predicted amino acid sequences. Cytosolic amino acid residues are highlighted in yellow, luminal amino acid residues are highlighted in light blue, amino acid residues in the putative pore loop are highlighted in dark blue. Acidic amino acid residues are indicated in red.
(PDF)

S4 File Raw data of this study. (XLSX)

S5 File Compilation of all Supporting Figures and Tables. (PDF)

S1 Table Oligonucleotides used in this study. (PDF)

S2 Table Predicted topology of TRPY1 and CgTRPF1 through 4. Topology prediction was performed with TOPCONS (http://topcons.net/) using standard settings and the full-length protein sequences of *S*. *cerevisiae* TRPY1, and CgTRPF1, CgTRPF2, CgTRPF3, and CgTRPF4.
(PDF)

S3 Table TRPF protein sequences used to generate the phylogenetic tree. Organisms were ordered by *phylum* and *class*. The sequenced *strain* and the *NCBI Taxid* of each species are given in the indicated columns. To link the proteins of this table with the tree, see column *TRPY1 Homologue No*. For the distinct identification of the proteins the *locus tag* may be used. *E-values* were calculated on NCBI.
(PDF)
