# Supplementary figures and images for: The Transient Receptor Potential (TRP) Channel Family in Colletotrichum graminicola: A Molecular and Physiological Analysis
Source: PLoS One. 2016 Jun 30;11(6):e0158561. doi: 10.1371/journal.pone.0158561 (PMC4928787; doi:10.1371/journal.pone.0158561)

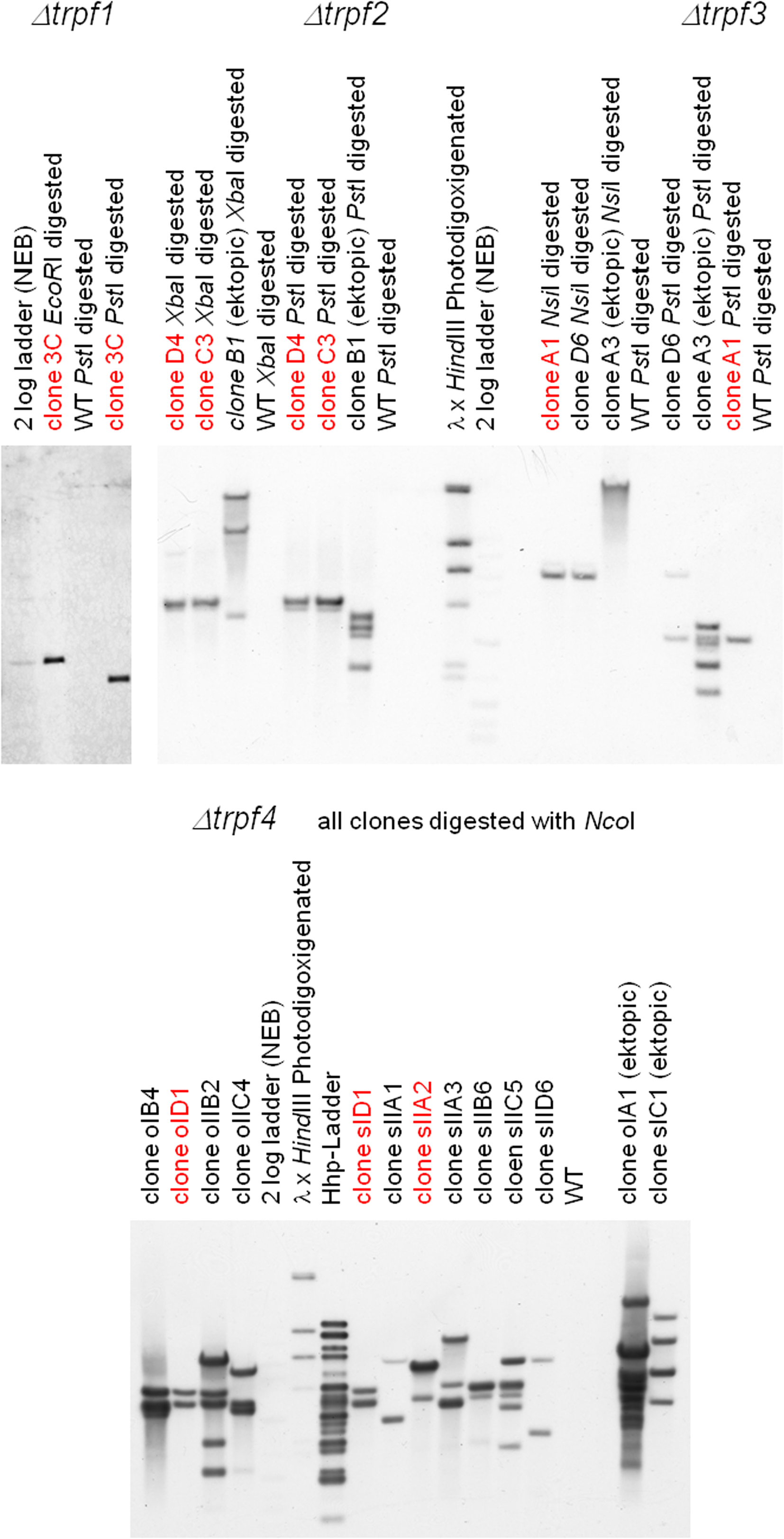

Supplement: S1 Fig — Genomic DNA was digested using the indicated restriction endonucleases and probed with a digoxigenin-labelled probe binding to the 5’ region of the hygromycinB phosphotransferase gene. Clones used in this study are indicated in red. (TIF) [file pone.0158561.s001.tif]

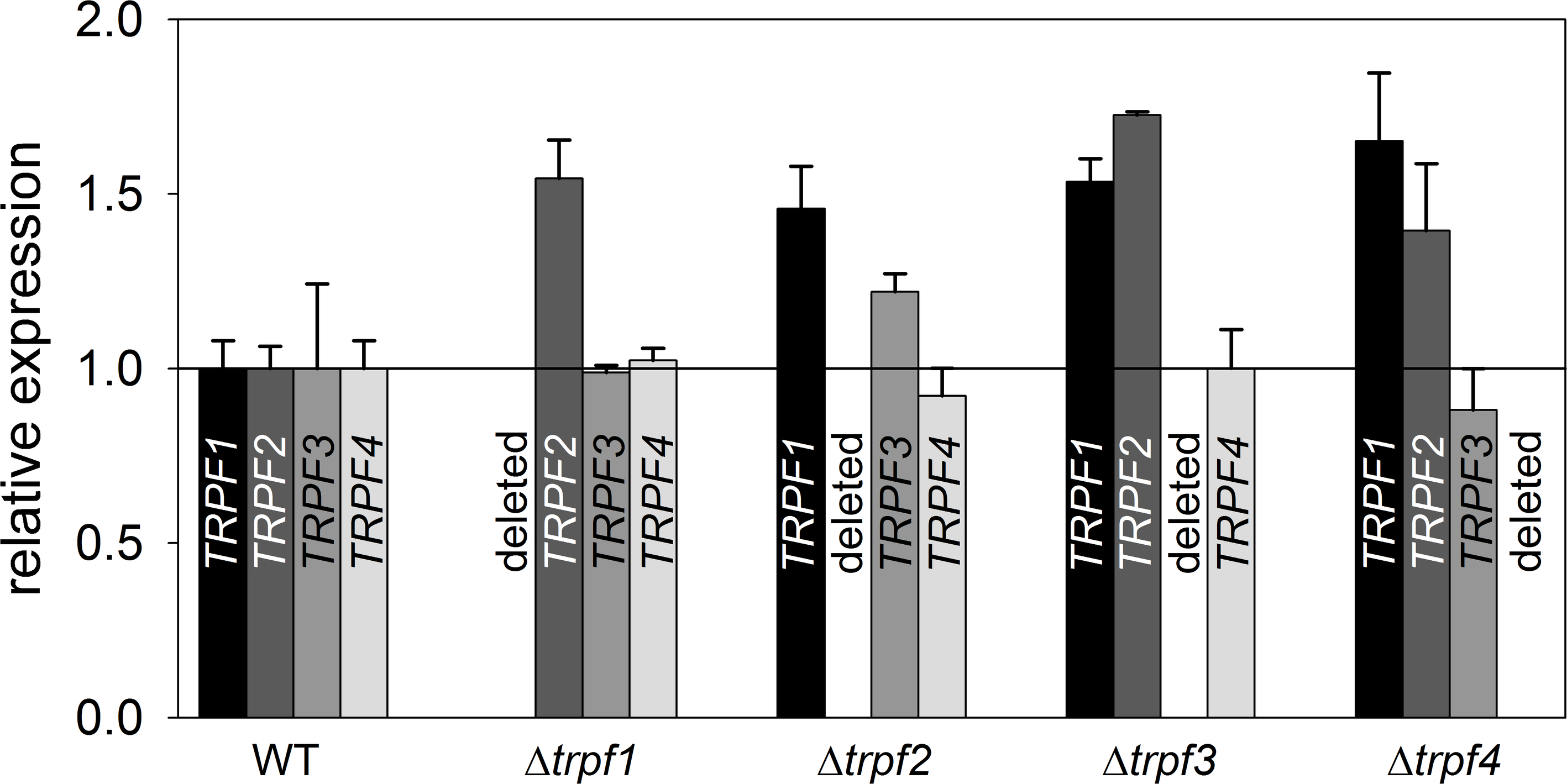

Supplement: S2 Fig — Strains were cultivated for 3.5 days on mLCM agar using the PAAP protocol [41] and assayed by qRT-PCR. Black: CgTRPF1, dark grey: CgTRPF2, middle grey: CgTRPF3, light grey: CgTRPF4. Data are means ± SE (N = 3). (TIF) [file pone.0158561.s002.tif]

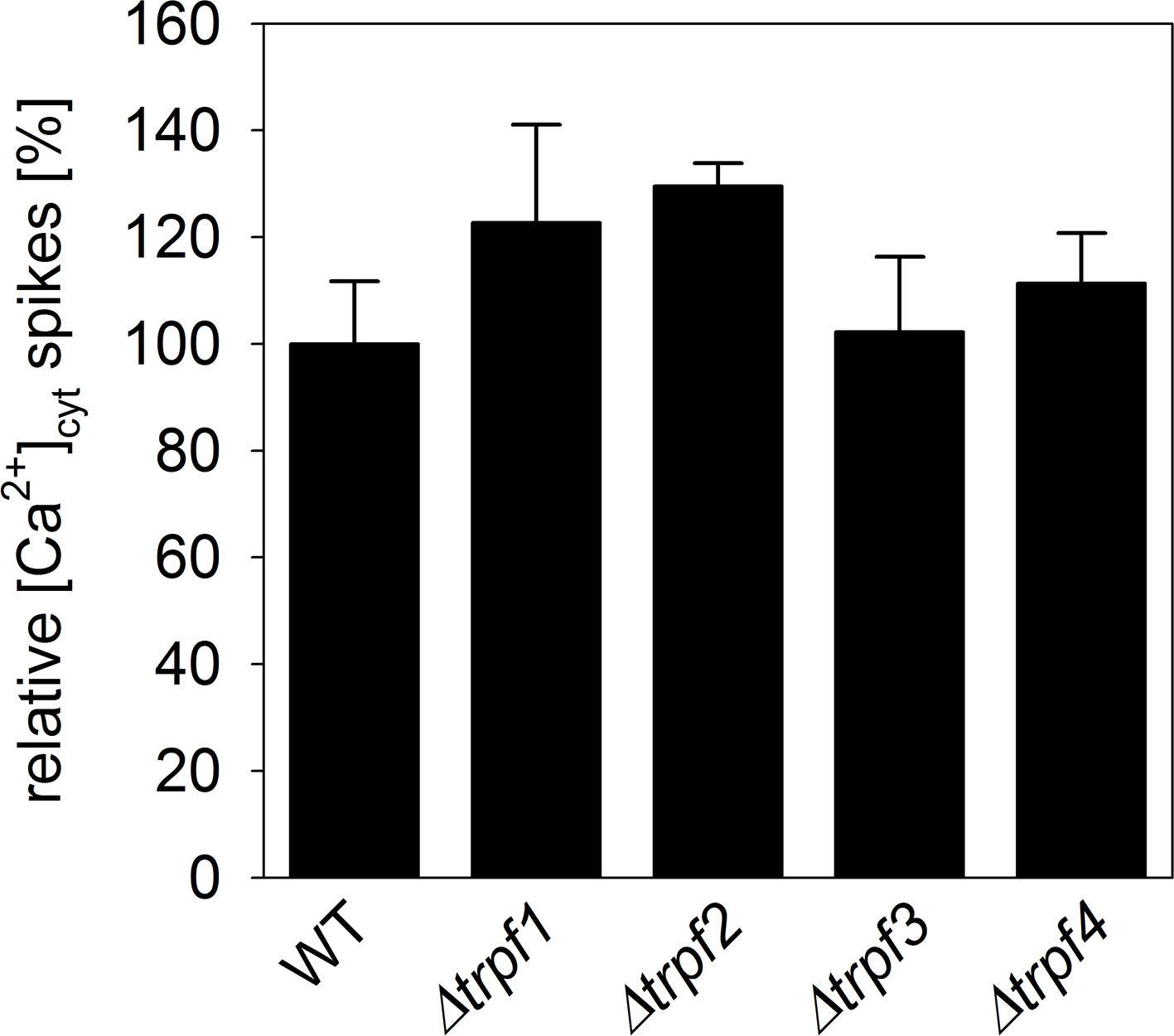

Supplement: S3 Fig — Colonies of C. graminicola wild type and Cgtrpf1 through 4 deletion strains expressing apoaequorin were grown for 80 h in 35-mm Petri dishes on mLCM agar supplemented with 10 μM coelenterazine. [Ca2+]cyt-dependent luminescence was detected for 20 min. Data are the means ± SE (N = 4). (TIF) [file pone.0158561.s003.tif]

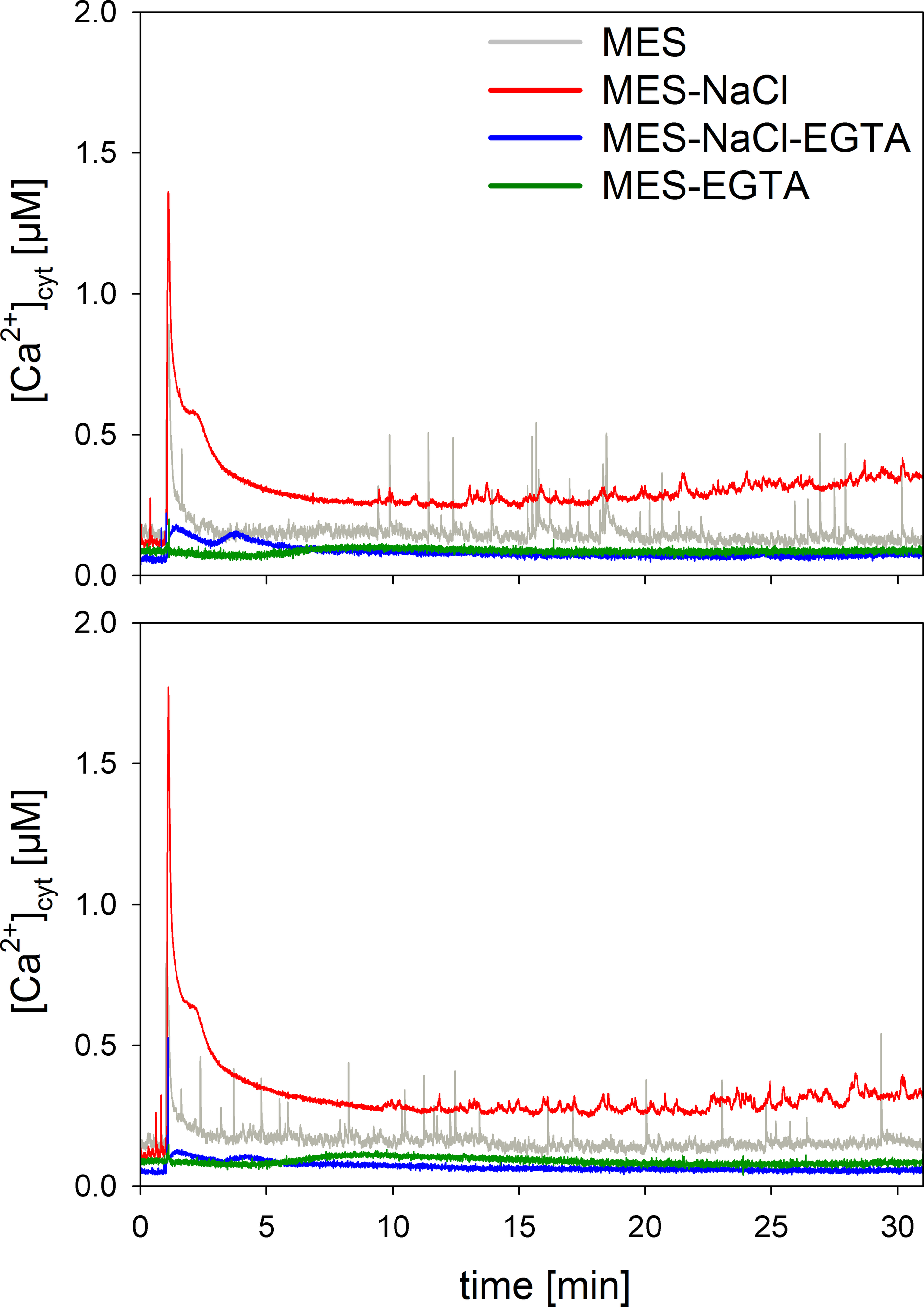

Supplement: S4 Fig — Whole colonies were pre-treated with 50 mM MES-KOH (pH 7.0) for 30 min prior to recording, followed by treatment with a solution (pH 7.0) containing 50 mM MES-KOH and no NaCl (grey line) or 1.5 M NaCl (final concentration; red line). To abolish the influx of extracellular Ca2+, colonies were pre-treated with a solution (pH 7.0) containing 50 mM MES-KOH and 25 mM EGTA for 30 min prior to measurement, followed by treatment with a solution (pH 7.0) containing 50 mM MES-KOH, 25 mM EGTA, and no NaCl (green line) or 1.5 M NaCl (final concentration; blue line). Treatment solutions were added after 1 min of measurement. Traces show single measurements in order to demonstrate [Ca2+]cyt spikes in the MES-KOH control treatment. (TIF) [file pone.0158561.s004.tif]

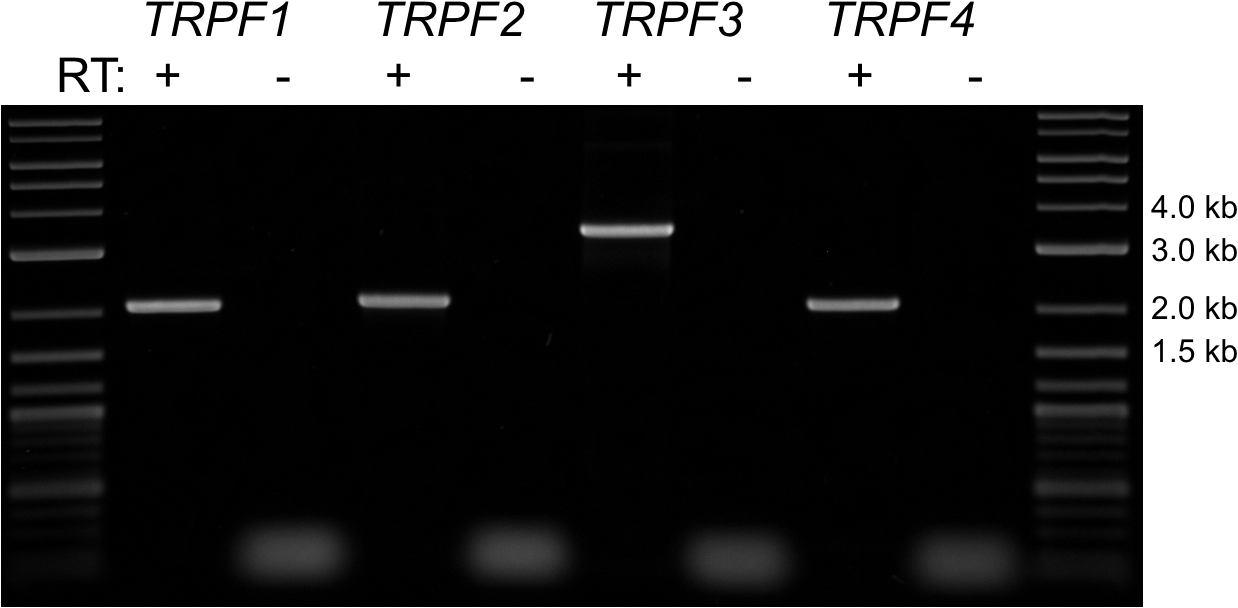

Supplement: S5 Fig — Full-length cDNAs of the TRPF genes were amplified from RNA extracted from log-phase cultures of S. cerevisiae trpy1Δ transformed with pFL61-CgTRPF1 through pFL61-CgTRPF4. Products were expected at 2098, 2152, 3522, and 2101 bp for CgTRPF1, CgTRPF2, CgTRPF3, and CgTRPF4, respectively. RT: + reverse transcriptase added in cDNA synthesis, − reverse transcriptase omitted in cDNA synthesis. (TIF) [file pone.0158561.s005.tif]
