# Supplementary material for: The Transient Receptor Potential (TRP) Channel Family in Colletotrichum graminicola: A Molecular and Physiological Analysis
Source: PLoS One. 2016 Jun 30;11(6):e0158561. doi: 10.1371/journal.pone.0158561 (PMC4928787; doi:10.1371/journal.pone.0158561)
Supplement: S3 File — Cytosolic amino acid residues are highlighted in yellow, luminal amino acid residues are highlighted in light blue, amino acid residues in the putative pore loop are highlighted in dark blue. Acidic amino acid residues are indicated in red. (PDF) [file pone.0158561.s008.pdf]

>TRPY1

MVSANGDLHLPISNEQCMPENNGSLGFEAPTPRQILRVTLNLKYLIDKVVPIVYDPNDIVCDHSEILS
PKVVKLAYEACGGNPKDKANKRKYQSVIIFSLLKVCEWYSILATMEVHNAKLYETRNLASQQLCKLLI
EREETRDLQFLFMQLLLRRYVINEN**DED**QEPLNALELATDMHCTTVIGSSGFQRCLKWIWRGWIVQNG
LDPTTFIKDDSLAEVSLISHFNPVRLKAPVYQNYLQMIFSFLFLGLYTLVVNGKDSERVQSFDLLESI
FYVFNTGFILDELTKLYYIGYAHLSFWNLFNDTTYLIITFAMGFRAMSVTPLNAKYSSEDWDKISYRV
LSCAAPFVWSRLLLYLESQRFIGIMLVILKHMMKESIVFFFLLFLIMIGFTQGFLGLDSADGKRDITG
PILGNLTITVLGLGSFDVFEEFAPPYAAILYYGYYFIVSVILLNILIALYSTAYQKVIDNA**DDE**YMAL
MSQKTLRYIRAP**DED**VYVSPLNLIEVFMTPIFRILPPKRAKDLSYTVMTIVYSPFLLLISVKETREAR
RIKYNRMKRLNDDANEYDTPWDLTDGYL***DDDD***GLFSDNRNSGMRATQLKNSRSLKLQRTAEQEDVHFK
VPKKWYKNVKKCSPSFEQYDNDDT**EDD**AGEDKDEVKELTKKVENLTAVITDLLEKLDIKDKKE

>TRPF1

MAAFNWVSRMLGWDRHSRHHHDFHSDWIRDDRRRLLPQYRSEDLESAIPAPEVTKIALKLRHLVELAV
PCELDEDDITKAHSTIITKKVIKAAKEAGGSHYGSCVVFCLIVCKRWFKHQALTELWDADLHRVRAVA
CEVIAKQIIEG**EED**LQYLMHSVLLRRYSIIVDGKSTPPANVIEKAVDLHAVRVIGSSGYQKCISYLWK
GWLVQDENDPSVFVDYKDKTNPSFLVHMDPDRIRAPMYQNATQVIISLVYIGLYTAVINSVNAKGVLD
SAEVLLYIFTLGFICEEVTKFWKAGYHILGFWNAFNGILYSFISLSLILRIIGLTHGEGEDARKYYSE
LSYNFLAFSAPMIWSRLLLYLDSFRFFGAMLVVLKVMMKESIIFFALLAVLIIGFLQAFVGLDLADDL
VAEDITFIMSAMANAIMQSPDFSGFDKFSPPFGIILYYCFTFIVMVVLLNILIALYNSAYEDIYDNAN
DEYLTLFAQKTMQFVRAPDENVYIPPFNLIEMVVIGLFWWMEKSKFERMSDFIMGFIYSPVLVFAAVF
ETRSAAEIRSNRARG**EEDDD**TIEEWEQMMDQVDFESDGWNKVCITAKTDLDVDPTISEVQKLRSEVEE
LKKMLVDISKAVSAGNAGNTQATNNLIDLGETTAPAAAGGSDKNKKRSKKGNKNKNNQQPTGDDGKAP
GGSSSS**DEE**

>TRPF2

MEEAECPRTPDLIDWSRPLSPWADENPATRTGDANGGMDPDLEADRHVSASATGTVSTEVPPSVYFTI
HRIRRLVLASIDDPYTLDQLREPRMNLLVVRPLVDRLYDPDDPAVVYCLFANRIQFLRQHTSTARQTV
NVARATLCELVASRVLRRYHEDHPGQIGLLILAHILVEGFDPFQGAPEEVESECRQLQWPIQRRDGHE
RKLTTLELAIISESKLFLSSSACQRLVDAVWNGVVTYTPLSFVDILPDHYEYASVSLYEPHQAPLLDV
GRLLVPWIRQMIELFQFITLVILYILTMVNRSSPTLSGWECVFAIYTAGWTLNEFAAIIEHGWVVHSQ
TLWSFLDITFSLIFSCYIFARIYDTMGYQGVVADGYGVHILCVAAPVLLTRLAFTLLPDNIVFIAMHA
MMKDFTRLTFISIWCFMGFFLALHWLVGSNDDTSAVAGGKAALTWYETCKWLIWTWFGLDGTGIDRSD
EFHPILGPALMIAFAFLGNILFLTILVAILSNTFSKLISDAPAEIQFRRAVLTFAGVKSDSIFSYPPP
FNLLALAALLPLKSMLSPRSFHDVNTSLIRALNAPALMIISALERRRVAHPRRARSQSLLNWSFSGFN
PHGDIQAVFKVEPPPAVERELEELDNLSDVGFAESELGDREPWDVGRSMPRRRPVRRRRPARMGSPIS
IVFPLPSVQRAGTNEFEPLSGSPVRRL

>TRPF3

MFSSLLRPKQSNRSRRVDRFHDRQSPSPGPAYRHYVGEPPRRATGDFTEGEDDQ**EEEDEEDD**GSGHSG
DDNEHV**DDED**LSRRSMPVLPLFSSTYLDSLPIYSITHAIRVIVQARTETTLTWDQIRSPQVSQFLVKP
MQQQIRTQQFNRATLYCLMANCLQFNKEGHMYPGNAGTSYTRALVCELLALKLLKEYNTRELIDALSY
DFYPLQGLPGAQPASTPKADPRNKARLTASRTSTLEVAIRASAKHFLAHPLVVQQLEAIWNGAISFYS
SADSLHREPPPSPTGIRPDARTPLLGGHNQKEGQQQSAPGRRSVILYDPRQASLFKLSRLRVPRYRFF
LSTMSLLVLIGLFLAVLSQRSARISSLELIFWFWSAGFMLDELVGFNEQGFSLYIMSFWNAFDLGILL
LLIVYYFMRVYGVFLVDAKHWNDSAYDVLAANAILLLPRIFSVLDHYQYFSQLLIAFRLMAVDLAAVC
ILILICCSGFFVFFTLADTNNDAGDIAFRIFQILMGFTPAAWEVWPGYNWLERGLMAFFLILCHFVIV
TILITVLTNSFMKIASNAREEHQFLFAINTISMVKNDALFSYVAPGNIFAWLLMPMRYCMPLKQFVKL
NRTIIKVTHFPLLFCIFLYEKYWLAPSMYEPTDLVENHHHHGRDRTISFADPASRPALFSPNIRVREE
SVAGYQKDHALEEVFRRGPGFATLRNQRRNERKKTQHAVRNWMDRNDEASASPQLSQWPTLDGSQGVR
PDWLRRLSLSRELRTRRSRQMSDVRSAASDPADLISQPAIGSFFRGIAAATTAK**DEE**NQQTDADG**DDE**
LVTN**DEEEEED**IATNADDSRHGPVIHQTSIDDYFNTTPSTKRFVPPTSSSLGSSGRPSVSATPRSARR
VPHSRTMSTNTILYAPQELRRPPSSSTHSPDPPPTRARVQNGRPAVVEPAVASGHRSPRRQTYVATTK
PRPIMPPRDAAQTAPNRAGLVSVDQRPRPNPLRRLSSVDLSVLSDNIVPDDPNGGGISSSFQTQMAMA
LMKDARLGGAGRGTDSGDSNRMSKLVLARMKTLEESFADVAKELRELKTTSSSTAPTTRRNSSGEELQ
KMTSLMSAEATRHKDGGSERPKLGGRRTTGKRPASRKSLLDPKSERARGKGKEPALSSGDDSDSESMS
PIKRSSM

>TRPF4

MPIRVRSKKLPKPRDLSPVSLHFDSLELPDIDTDDTFRNVVKKLSVYFVDVIVLPSTFEQLRTTAAGE
PLRVLVDHLNATCTNPAIVNALLALKWHYAVDESESNKGLCEARASACEIVAWRFLTRLSEREAVDYC
LYEIPDPKEPPTPPPNADLESNEDSPLLSGGSWSGNGSRRSSVRPGSSAKRYQLLSSLSRLTMSMHAD
Q**EEEEDDD**PTSPFTNLNALEIAAVADAKRFLSQHVVQKIITGIWNGDIIFWDSLSVHAQKKPRFYNAH
TTDIFSRLRVPKYLKAWEAFFFFLFLCLYYSVLVEQNTERITHNEIVLYIWLAAFLYDELSEWADAGT
IFYATDVWNLFDMIMIGIGIAFAVLRVIGIANNDRHITNTAFNVFALEALFMVPRVFSLLSLSPYWGT
LIPCLKEMGKDFVKFMVLVVVIYLGFLTTFSLIGQDAYNFSHMTMILTKIFFGSAYVGIEIMDDIDKV
FGPPLMIIFIILSSFLLMGSLTGMLSNSFSRVITHAREEYLYVYSVYVLEASTSNRLTHFYPPFNVIA
LVIFRPLRLFLPSDDKFRQSRILLLKATHLPIVGLIRMYESIRRRVMPDEYAGFKGPRGATGATRQRS
RGAFQANRPSSGYRPARSPRPEERLESRHSSRPQHRAIEADEADAPSAVEVRISELNDKIDKLTALVE
ALQQRPGSP

Key:

MVSANGDLHL cytosolic

PSDDKFRQSR luminal

IGIGIAFAVL transmembrane domain

IGQDAYNFSH putative pore loop
