# Supplementary material for: The Transient Receptor Potential (TRP) Channel Family in Colletotrichum graminicola: A Molecular and Physiological Analysis
Source: PLoS One. 2016 Jun 30;11(6):e0158561. doi: 10.1371/journal.pone.0158561 (PMC4928787; doi:10.1371/journal.pone.0158561)
Supplement: S5 File — (PDF) [file pone.0158561.s010.pdf]

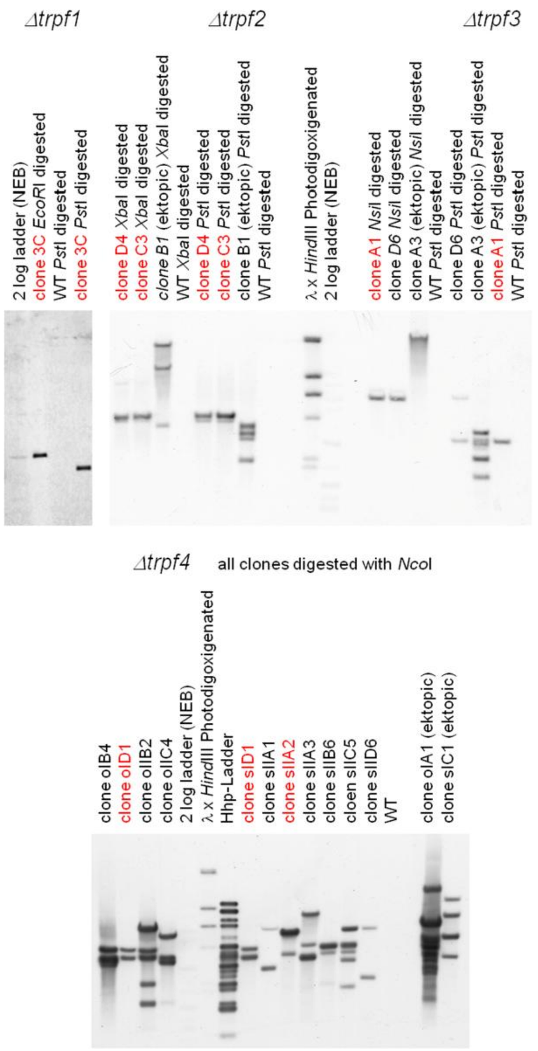

S1 Fig. Genomic Southern Blots of the deletion mutants for *CgTRPF1* through *4*. Genomic DNA was digested using the indicated restriction endonucleases and probed with a digoxigenin-labelled probe binding to the 5' region of the *hygromycinB phosphotransferase* gene. Clones used in this study are indicated in red.

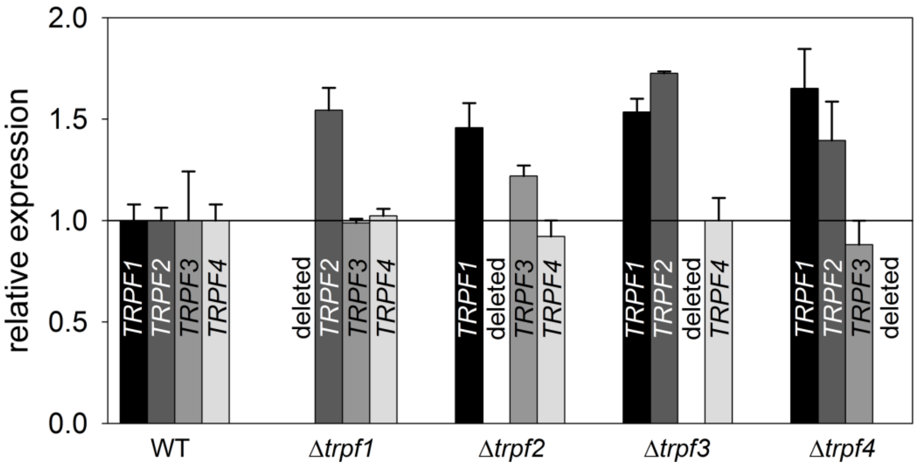

S2 Fig. Relative expression of the *CgTRPF* genes in *C. graminicola* wild type and in strains deleted for individual *CgTRPF* genes. Strains were cultivated for 3.5 days on mLCM agar using the PAAP protocol [41] and assayed by qRT-PCR. Black: *CgTRPF1*, dark grey: *CgTRPF2*, middle grey: *CgTRPF3*, light grey: *CgTRPF4*. Data are means ± SE (N=3).

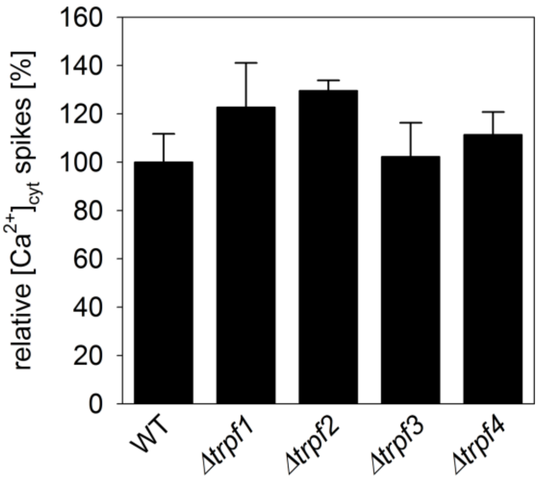

S3 Fig. Relative $[Ca^{2+}]_{cyt}$ spiking rate during colony growth of *C. graminicola*. Colonies of *C. graminicola* wild type and *Cgtrpf1* through *4* deletion strains expressing apoaequorin were grown for 80 h in 35-mm Petri dishes on mLCM agar supplemented with 10 µM coelenterazine. $[Ca^{2+}]_{cyt}$-dependent luminescence was detected for 20 min. Data are the means ± SE (N=4).

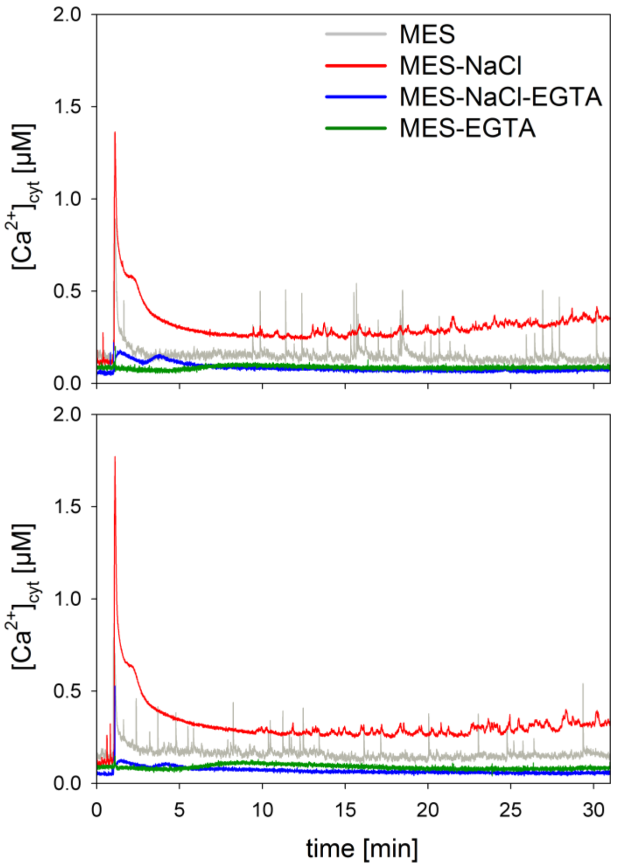

S4 Fig. Additional repeats of the aequorin luminescence measurements of $[Ca^{2+}]_{cyt}$ responses of *C. graminicola* to NaCl. Whole colonies were pre-treated with 50 mM MES-KOH (pH 7.0) for 30 min prior to recording, followed by treatment with a solution (pH 7.0) containing 50 mM MES-KOH and no NaCl (grey line) or 1.5 M NaCl (final concentration; red line). To abolish the influx of extracellular Ca^{2+}, colonies were pre-treated with a solution (pH 7.0) containing 50 mM MES-KOH and 25 mM EGTA for 30 min prior to measurement, followed by treatment with a solution (pH 7.0) containing 50 mM MES-KOH, 25 mM EGTA, and no NaCl (green line) or 1.5 M NaCl (final concentration; blue line). Treatment solutions were added after 1 min of measurement. Traces show single measurements in order to demonstrate $[Ca^{2+}]_{cyt}$ spikes in the MES-KOH control treatment.

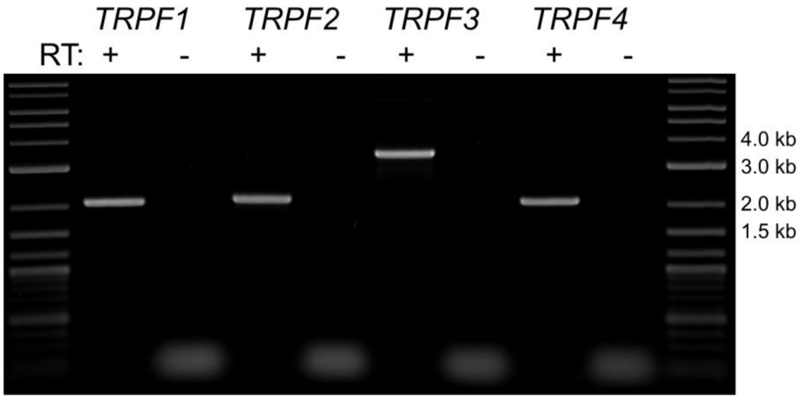

S5 Fig. Expression of *CgTRPF* genes in transformed *S. cerevisiae trpy1*Δ strains. Full-length cDNAs of the *TRPF* genes were amplified from RNA extracted from log-phase cultures of *S. cerevisiae trpy1*Δ transformed with pFL61-CgTRPF1 through pFL61-CgTRPF4. Products were expected at 2098, 2152, 3522, and 2101 bp for *CgTRPF1*, *CgTRPF2*, *CgTRPF3*, and *CgTRPF4*, respectively. RT: + reverse transcriptase added in cDNA synthesis, - reverse transcriptase omitted in cDNA synthesis.

S1 Table. Oligonucleotides used in this study.

Purpose	Name	Sequence	Ref.
RACE	TRPF1_3'RACE	TGGGTTTCATCTACTCGCCTGTCCTCG	
	TRPF1_5'RACE	GCCGATGTAGACCAGGGAGATGATGACC	
	TRPF2_3'RACE	CCACCCCCATTCAACCTCCTTGCTCT	
	TRPF2_5'RACE	GCGGTGCCTGGTGCGGTTCATAAAG	
	TRPF2_5'RACE_2	GCGAAGAGGCAGTAGACGACAGCAG	
	TRPF3_3'RACE	CCAACGCAGCACCATCCTCATCACGG	
	TRPF3_5'RACE	GATACGGGGAAGCAGCAGGATGGC	
	TRPF3_5'RACE_2	AGTGCTTTGCGTCTACGAGGAATACCC	
	TRPF4_3'RACE	CGAGCAGAACACCGAACGCATCACCCAC	
	TRPF4_5'RACE	CGTGTGGGCGTTGTAGAAGCGGGGC	
	TRPF4_5'RACE_2	GGCTTCGCAGAGACCCTTGTTGGAC	
qRT-PCR	Real_TRPF1_for	CCGACTTTAGCGGGTTCGAC	
	Real_TRPF1_rev	TGTCCTCGTACGCCGAGTTG	
	Real_TRPF2_for	CGGATTCGGCGTCTCGTA	
	Real_TRPF2_rev	CAGGGTCGTCGGGGTCAT	
	Real_TRPF3_for	CTTGTCTTGGCACGGATGAA	
	Real_TRPF3_rev	GCCTTGTGGTGGGAGCAGT	
	Real_TRPF4_for	TGTTCTTAGGGTCATCGGTATTG	
	Real_TRPF4_rev	GGATTAGCGTGCCCCAGTAG	
	Real_Act_for	TCCTACGAGCTTCCTGACGG	*
	Real_Act_rev	CCGCTCTCAAGACCAAGGAC	*
	Real_HistH3_for	CGAGATCCGTCGCTACCAGA	*
	Real_HistH3_rev	GGAGGTCGGACTTGAAGTCCT	*
	Real_ILV5_for	GCTGCCTCATGGGTGGTATC	
	Real_ILV5_rev	CCTCGACGGTCTCGTTGAAG	
Subcellular localization	TRPF1_Loc_for	TTAATTAACTCTTCGGGGTGTTCCTGTGTG	
	TRPF1_Loc_rev	GGCCGGCC*CC*CTCTTCGTCACTCGAGCTAGAGCC	
	TRPF2_Loc_for	AAAAAATTAATTAACGGTGTTACCAGCAAGACAAGTGAG	
	TRPF2_Loc_rev	AAAAAAGGCCGGCC*CC*CAGCCTCCTTACAGGTGACCCAG	
	TRPF3_Loc_for	AAAAAATTAATTAACCTGACAACGGCGACGATGG	
	TRPF3_Loc_rev	AAAAAAGGCCGGCC*CC*CATCGAGCTCCGTTTAATCGGC	
	TRPF4_5'Flank/Loc_f	AAAAAATTAATTAAACAGTAGTTGGGGAGAGACATTACG	
	TRPF4_Loc_rev	AAAAAAGGCCGGCC*CC*AGAGGGCGATCCGGGC	
Generation of deletion cassettes	uni-hyg.F1	TGTACGACTGTCAGTTGCACTGACCGGTGCCTGGATCTTC	#
	uni-hyg.R1	AAGCTAGTGAGACTCCAGACGGTCGGCATCTACTCTATTCC	#
	TRPF1_5'Flank_for	GAGTTGATTGGATGGGACGATG	
	TRPF1_5'Flank_rev	GTGCAACTGACAGTCGTACACCTTCCTATGCTATGGGCGAC	
	TRPF1_3'Flank_for	TCTGGAGTCTCACTAGCTTGACCCGTGCTGAATGATGTG	
	TRPF1_3'Flank_rev	GTCTCACTTTCACTCTTCCACTCG	
	TRPF1_5'Flank_nest	GGTCTGGCACTGAAGTAGCAATAAC	
	TRPF1_3'Flank_nest	TTGCTTCGGGCTATTGATGG	
	TRPF2_5'Flank_for	GAACCAACCTTAGAAGAATGTCGG	
	TRPF2_5'Flank_rev	GTGCAACTGACAGTCGTACAGAAGTCGGGATTTGATGATGGTAG	
	TRPF2_3'Flank_for	TCTGGAGTCTCACTAGCTTCAACGCCAGCATCTCCAATAC	
	TRPF2_3'Flank_rev	GGTCTGCTCTTTCGTAGTGTTCTTC	
	TRPF2_5'Flank_nest	AACCTTCTCAGCATCCATTCTCTATC	

	TRPF2_3'Flank_nest	AGCACTTTGGAGTATCTTTGGCAG
	TRPF3_5'Flank_for	TGCCTTGCCTTGCCGTG
	TRPF3_5'Flank_rev	GTGCAACTGACAGTCGTACAGCTTTTGCCTACAGGAGAATGG
	TRPF3_3'Flank_for	TCTGGAGTCTCACTAGCTTGGGTTACGCCTGGAGCATTGTTC
	TRPF3_3'Flank_rev	CCCTCCACACCCCCGAGAATC
	TRPF3_5'Flank_nest	TAGCCTCGTCTTGCCTCCTTG
	TRPF3_3'Flank_nest	CATCGTAGAACACCGCAGATAACC
	TRPF4_5'Flank/Loc_f	*AAAAAATTAATTAA*ACAGTAGTTGGGGAGAGACATTACG
	TRPF4_5'Flank_rev	GTGCAACTGACAGTCGTACAACGGGAAGAAGAGGGCGAC
	TRPF4_3'Flank_for	TCTGGAGTCTCACTAGCTTATGATGGTTCTTGAAAAGGTAGATTGC
	TRPF4_3'Flank_rev	AGGCTATGCGATGACTGTCTCACTTA
	TRPF4_5'Flank_nest	GTTTTGTTGTAGACTGCGACGG
	TRPF4_3'Flank_nest	TCTCAGCCAATCCAAGCCAC
Southern Blot	Hph-5'-South-for	CTAAAATCCGCCGCCTCCAC
Probe synthesis	Hph-5'-South-rev	CGGACAGACGGGGCAAAGC
Yeast	TRPF1_Yeast_for	AAAAAAGCGGCCGCATGGCCGCCTTCAACTGGG
Comple-	TRPF1_Yeast_rev	AAAAAAGCGGCCGCTCACTCTTCGTCACTCGAGCTAGAGC
mentation	TRPF2_Yeast_for	AAAAAAGCGGCCGCATGGAAGAAGCCGAATGTCCG
	TRPF2_Yeast_rev	AAAAAAGCGGCCGCTCACAGCCTCCTTACAGGTGACC
	TRPF3_Yeast_for	AAGCGGCCGCATGTTTTCCTCCCTGCTGCG
	TRPF3_Yeast_rev	AAAAAAGCGGCCGCTTACATCGAGCTCCGTTTAATCG
	TRPF4_Yeast_for	AAAAAAGCGGCCGCATGCCCATCCGCGTCAGGT
	TRPF4_Yeast_rev	AAAAAAGCGGCCGCGATGTGATGTATTTTATTCCCAGTTCGC
	TRPY1_Yeast_for	AAAAAAGCGGCCGCATGGTATCAGCCAACGGCG
	TRPY1_Yeast_rev	AAAAAAGCGGCCGCTTACTCTTTCTTATCCTTTATGTCTAATTTTTC

Underlined regions in the oligonucleotides for subcellular localization indicate restriction enzyme sites for *Pac*I and *Fse*I.

Italicized CC in reverse oligonucleotides for subcellular localization indicate a part of a five amino acid linker between the *TRPF* genes and the fluorescent tag not covered by the *Fse*I site.

Underlined regions in the oligonucleotides for the generation of deletion cassettes indicate universal overhangs for fusion PCR.

Italicized region in the dual purpose oligonucleotide TRPF4_5'Flank/Loc_f indicates the part needed for subcellular localization only.

Underlined regions in the oligonucleotides for Yeast complementation indicate restriction enzyme sites for *Not*I.

References:

* Krijger J-J, Horbach R, Behr M, Schweizer P, Deising HB, Wirsel SGR. The yeast signal sequence trap identifies secreted proteins of the hemibiotrophic corn pathogen *Colletotrichum graminicola*. Mol. Plant Micr. Interact. 2008; 21: 1325-1336.

Abou Ammar G, Tryono R, Döll K, Karlovsky P, Deising HB, Wirsel SGR. Identification of ABC transporter genes of *Fusarium graminearum* with roles in azole tolerance and/or virulence. PLOS ONE 2013; 8: e79042

S2 Table. Predicted topology of TRPY1 and CgTRPF1 through 4. Topology prediction was performed with TOPCONS (http://topcons.net/) using standard settings and the full-length protein sequences of *S. cerevisiae* TRPY1, and *C. graminicola* TRPF1, TRPF2, TRPF3, and TRPF4.

	amino acid position					element Size				
	TRPY1	TRPF1	TRPF2	TRPF3	TRPF4	TRPY1	TRPF1	TRPF2	TRPF3	TRPF4
**cytosol**	**001 - 234**	**001 - 241**	**001 - 280**	**001 - 336**	**001 - 285**	**234**	**241**	**280**	**336**	**285**
**TM1**	**235 - 255**	**242 - 262**	**281 - 301**	**337 - 357**	**286 - 306**	**21**	**21**	**21**	**21**	**21**
**vesicular**	**256 - 265**	**263 - 269**	**302 - 309**	**358 - 364**	**307 - 311**	**10**	**7**	**8**	**7**	**5**
**TM2**	**266 - 286**	**270 - 290**	**310 - 330**	**365 - 385**	**312 - 332**	**21**	**21**	**21**	**21**	**21**
**cytosol**	**287 - 298**	**291 - 303**	**331 - 342**	**386 - 397**	**333 - 349**	**12**	**13**	**12**	**12**	**17**
**TM3**	**299 - 319**	**304 - 324**	**343 - 363**	**398 - 418**	**350 - 370**	**21**	**21**	**21**	**21**	**21**
**vesicular**	**320 - 335**	**325 - 341**	**364 - 376**	**419 - 431**	**371 - 380**	**16**	**17**	**13**	**13**	**10**
**TM4**	**336 - 356**	**342 - 362**	**377 - 397**	**432 - 452**	**381 - 401**	**21**	**21**	**21**	**21**	**21**
**cytosol**	**357 - 375**	**363 - 380**	**398 - 412**	**453 - 466**	**402 - 419**	**19**	**18**	**15**	**14**	**18**
**TM5**	**376 - 396**	**381 - 401**	**413 - 433**	**467 - 487**	**420 - 440**	**21**	**21**	**21**	**21**	**21**
**putative pore region**	**397 - 436**	**402 - 442**	**434 - 484**	**488 - 529**	**441 - 479**	**40**	**41**	**51**	**42**	**39**
**TM6**	**437 - 457**	**443 - 463**	**485 - 505**	**530 - 550**	**480 - 500**	**21**	**21**	**21**	**21**	**21**
**cytosol**	**458 - 489**	**464 - 526**	**506 - 535**	**551 - 612**	**501 - 534**	**32**	**63**	**30**	**62**	**34**
TM7	490 - 510	527 - 547	536 - 556	613 - 633	535 - 555	21	21	21	21	21
vesicular	511 - 519	548 - 689	557 - 567	634 - 1163	556 - 565	9	142	11	530	10
TM8	520 - 540	not exsistent	568 - 588	not exsistent	566 - 586	21		21		21
cytosol	541 - 675	not exsistent	589 - 707	not exsistent	587 - 689	135		119		103

S3 Table. TRPF protein sequences used to generate the phylogenetic tree. Organisms were ordered by *phylum* and *class*. The sequenced *strain* and the *NCBI Taxid* of each species are given in the indicated columns. To link the proteins of this table with the tree, see column *TRPY1 Homologue No*. For the distinct identification of the proteins the *Locus Tag* may be used. *E-values* were calculated on NCBI.

Phylum	Class	Species	Strain	NCBI Taxid	TRPY1 Homo-loge No.	E-value	Locus tag	Source
Ascomycota	Dothideomycetes	*Mycosphaerella graminicola*	IPO323	336722	1	2e-141	MYCGRDRAFT_76604	NCBI
					2	2e-15	MYCGRDRAFT_20435	
					4	4e-12	MYCGRDRAFT_75841	
		Pyrenophora tritici-repentis	Pt-1C-BFP race 1	426418	1	4e-134	PTRG_05238.1	Broad institute [1)]
					2	9e-12	PTRG_06414.1	
					3	9e-16	PTRG_08905.1	
	Eurotiomycetes	*Aspergillus fumigatus*	Af293	330879	1	1e-131	Afu3g13490	NCBI
					3	8e-19	Afu6g11300	
		Penicillium chrysogenum	Wisconsin 54-1255	500485	1	6e-122	Pc13g15030	NCBI
					3	3e-24	Pc21g21930	
	Leotiomycetes	*Botrytis cinerea*	B05.10	40559	1	1e-137	BC1T_03504	Broad institute [1)]
					3	1e-15	BC1T_03018	
	Pezizomycetes	*Tuber melanosporum*	Mel28	656061	1	1e-138	GSTUM_00011244001	NCBI
	Saccharomycetes	*Candida albicans*	SC5314	237561	1 TRPY3 [2)]	0	CaO19.2209	NCBI
		Debaryomyces hansenii	CBS767	284592	1	0	DEHA2C08228g	NCBI
		Lodderomyces elongisporus	NRRL YB-4239	379508	1	0	LELG_01988	NCBI
		Saccharomyces cerevisiae	S288c	559292	1 TRPY1 [3)]	0	YOR087W	NCBI
		Zygosaccharomyces rouxii	CBS 732	559307	1	0	ZYRO0C15510g	NCBI
	Sordariomycetes	*Colletotrichum graminicola*	M1.001	31870	1 TRPF1 [4)]	3e-138	GLRG_09114.1	NCBI
					2 TRPF2 [4)]	2e-17	GLRG_10771.1	
					3 TRPF3 [4)]	6e-18	GLRG_08368.1	
					4 TRPF4 [4)]	9e-14	GLRG_09848.1	
		Colletotrichum higginsianum	IMI 349063	80884	1	6e-135	CH063_11552.1	NCBI
					2	3e-10	CH063_03277.1	
					3	1e-18	CH063_02273.1	
					4	1e-15	CH063_07357.1	
		Fusarium graminearum	PH-1	5518	1 TRPGz [5)]	1e-123	FGSG_04178.3	NCBI
					4	2e-15	FGSG_05259.3	
		Magnaporthe oryzae	70-15	242507	1 [6)]	2e-137	MGG_09828.6	NCBI
					3	4e-16	MGG_01538.6	
					4	1e-21	MGG_06118.6	
		Neurospora crassa OR74A	OR74A	367110	1	4e-106	NCU16725.7	Broad institute [1)]
					2	2e-16	NCU04465.7	
					3	5e-13	NCU08283.7	
					4	2e-16	NCU06601.7	
Basidiomycota	Agaricomycetes	*Laccaria bicolor*	S238N-H82	486041	1	1e-53	LACBIDRAFT_311591	NCBI
					2a	5e-11	LACBIDRAFT_305275	
					2b	5e-08	LACBIDRAFT_297283	
	Pucciniomycetes	*Puccinia triticina*	BBBD Race 1	630390	2	1e-9	PTTG_08032T0	Broad institute [1)]
	Tremellomycetes	*Cryptococcus neoformans*	grubii H99	235443	1	2e-54	CNAG_07368.2	NCBI
					2	8e-07	CNAG_03844.2	
	Ustilaginomycetes	*Ustilago maydis*	521	237631	1	4e-67	UM03685.1	NCBI
					2	1e-12	UM04857.1	

[1] In the cases where no appropriate NCBI database entry was available, *E-values* were calculated locally using appropriate databases downloaded from *Broad Institute* as indicated in column *Source*.

[2] Zhou XL, Loukin SH, Coria R, Kung C, Saimi Y. Heterologously expressed fungal transient receptor potential channels retain mechanosensitivity in vitro and osmotic response *in vivo*. Eur. Biophys. J. 2005; 34: 413-422.

[3] Palmer CP, Zhou XL, Lin J, Loukin SH, Kung C, et al. A TRP homolog in *Saccharomyces cerevisiae* forms an intracellular Ca^{2+}-permeable channel in the yeast vacuolar membrane. Proc. Natl. Acad. Sci. USA 2001; 98: 7801-7805.

[4] this study

[5] Ihara M, Hamamoto S, Miyanoiri Y, Takeda M, Kainosho M, et al. Molecular bases of multimodal regulation of a fungal transient receptor potential (TRP) channel. J. Biol. Chem. 2013; 288: 15303-15317.

[6] Nguyen QB, Kadotani N, Kasahara S, Tosa Y, Mayama S, et al. Systematic functional analysis of calcium-signalling proteins in the genome of the rice-blast fungus, *Magnaporthe oryzae*, using a high-throughput RNA-silencing system. Mol. Microbiol. 2008; 68: 1348-1365.
