# Supplementary material for: The Transient Receptor Potential (TRP) Channel Family in Colletotrichum graminicola: A Molecular and Physiological Analysis
Source: PLoS One. 2016 Jun 30;11(6):e0158561. doi: 10.1371/journal.pone.0158561 (PMC4928787; doi:10.1371/journal.pone.0158561)
Supplement: S1 Table — (PDF) [file pone.0158561.s011.pdf]

S1 Table. Oligonucleotides used in this study.

Purpose	Name	Sequence	Ref.
RACE	TRPF1_3'RACE	TGGGTTTCATCTACTCGCCTGTCCTCG	
	TRPF1_5'RACE	GCCGATGTAGACCAGGGAGATGATGACC	
	TRPF2_3'RACE	CCACCCCCATTCAACCTCCTTGCTCT	
	TRPF2_5'RACE	GCGGTGCCTGGTGCGGTTCATAAAG	
	TRPF2_5'RACE_2	GCGAAGAGGCAGTAGACGACAGCAG	
	TRPF3_3'RACE	CCAACGCAGCACCATCCTCATCACGG	
	TRPF3_5'RACE	GATACGGGGAAGCAGCAGGATGGC	
	TRPF3_5'RACE_2	AGTGCTTTGCGTCTACGAGGAATACCC	
	TRPF4_3'RACE	CGAGCAGAACACCGAACGCATCACCCAC	
	TRPF4_5'RACE	CGTGTGGGCGTTGTAGAAGCGGGGC	
	TRPF4_5'RACE_2	GGCTTCGCAGAGACCCTTGTTGGAC	
qRT-PCR	Real_TRPF1_for	CCGACTTTAGCGGGTTCGAC	
	Real_TRPF1_rev	TGTCCTCGTACGCCGAGTTG	
	Real_TRPF2_for	CGGATTCGGCGTCTCGTA	
	Real_TRPF2_rev	CAGGGTCGTCGGGGTCAT	
	Real_TRPF3_for	CTTGTCTTGGCACGGATGAA	
	Real_TRPF3_rev	GCCTTGTGGTGGGAGCAGT	
	Real_TRPF4_for	TGTTCTTAGGGTCATCGGTATTG	
	Real_TRPF4_rev	GGATTAGCGTGCCCCAGTAG	
	Real_Act_for	TCCTACGAGCTTCCTGACGG	*
	Real_Act_rev	CCGCTCTCAAGACCAAGGAC	*
	Real_HistH3_for	CGAGATCCGTCGCTACCAGA	*
	Real_HistH3_rev	GGAGGTCGGACTTGAAGTCCT	*
	Real_ILV5_for	GCTGCCTCATGGGTGGTATC	
	Real_ILV5_rev	CCTCGACGGTCTCGTTGAAG	
Subcellular localization	TRPF1_Loc_for	TTAATTAACTCTTCGGGGTGTTCCTGTGTG	
	TRPF1_Loc_rev	GGCCGGCC*CC*CTCTTCGTCACTCGAGCTAGAGCC	
	TRPF2_Loc_for	AAAAAATTAATTAACGGTGTTACCAGCAAGACAAGTGAG	
	TRPF2_Loc_rev	AAAAAAGGCCGGCC*CC*CAGCCTCCTTACAGGTGACCCAG	
	TRPF3_Loc_for	AAAAAATTAATTAACCTGACAACGGCGACGATGG	
	TRPF3_Loc_rev	AAAAAAGGCCGGCC*CC*CATCGAGCTCCGTTTAATCGGC	
	TRPF4_5'Flank/Loc_f	AAAAAATTAATTAAACAGTAGTTGGGGAGAGACATTACG	
	TRPF4_Loc_rev	AAAAAAGGCCGGCC*CC*AGAGGGCGATCCGGGC	
Generation of deletion cassettes	uni-hyg.F1	TGTACGACTGTCAGTTGCACTGACCGGTGCCTGGATCTTC	#
	uni-hyg.R1	AAGCTAGTGAGACTCCAGACGGTCGGCATCTACTCTATTCC	#
	TRPF1_5'Flank_for	GAGTTGATTGGATGGGACGATG	
	TRPF1_5'Flank_rev	GTGCAACTGACAGTCGTACACCTTCCTATGCTATGGGCGAC	
	TRPF1_3'Flank_for	TCTGGAGTCTCACTAGCTTGACCCGTGCTGAATGATGTG	
	TRPF1_3'Flank_rev	GTCTCACTTTCACTCTTCCACTCG	
	TRPF1_5'Flank_nest	GGTCTGGCACTGAAGTAGCAATAAC	
	TRPF1_3'Flank_nest	TTGCTTCGGGCTATTGATGG	
	TRPF2_5'Flank_for	GAACCAACCTTAGAAGAATGTCGG	
	TRPF2_5'Flank_rev	GTGCAACTGACAGTCGTACAGAAGTCGGGATTTGATGATGGTAG	
	TRPF2_3'Flank_for	TCTGGAGTCTCACTAGCTTCAACGCCAGCATCTCCAATAC	

	TRPF2_3'Flank_rev	GGTCTGCTCTTTCGTAGTGTTCTTC
	TRPF2_5'Flank_nest	AACCTTCTCAGCATCCATTCTCTATC
	TRPF2_3'Flank_nest	AGCACTTTGGAGTATCTTTGGCAG
	TRPF3_5'Flank_for	TGCCTTGCCTTGCCGTG
	TRPF3_5'Flank_rev	GTGCAACTGACAGTCGTACAGCTTTTGCCTACAGGAGAATGG
	TRPF3_3'Flank_for	TCTGGAGTCTCACTAGCTTGGGTTACGCCTGGAGCATTGTTC
	TRPF3_3'Flank_rev	CCCTCCACACCCCCGAGAATC
	TRPF3_5'Flank_nest	TAGCCTCGTCTTGCCTCCTTG
	TRPF3_3'Flank_nest	CATCGTAGAACACCGCAGATAACC
	TRPF4_5'Flank/Loc_f	*AAAAAATTAATTAA*ACAGTAGTTGGGGAGAGACATTACG
	TRPF4_5'Flank_rev	GTGCAACTGACAGTCGTACAACGGGAAGAAGAGGGCGAC
	TRPF4_3'Flank_for	TCTGGAGTCTCACTAGCTTATGATGGTTCTTGAAAAGGTAGATTGC
	TRPF4_3'Flank_rev	AGGCTATGCGATGACTGTCTCACTTA
	TRPF4_5'Flank_nest	GTTTTGTTGTAGACTGCGACGG
	TRPF4_3'Flank_nest	TCTCAGCCAATCCAAGCCAC
Southern Blot	Hph-5'-South-for	CTAAAATCCGCCGCCTCCAC
Probe synthesis	Hph-5'-South-rev	CGGACAGACGGGGCAAAGC
Yeast	TRPF1_Yeast_for	AAAAAAGCGGCCGCATGGCCGCCTTCAACTGGG
Comple-	TRPF1_Yeast_rev	AAAAAAGCGGCCGCTCACTCTTCGTCACTCGAGCTAGAGC
mentation	TRPF2_Yeast_for	AAAAAAGCGGCCGCATGGAAGAAGCCGAATGTCCG
	TRPF2_Yeast_rev	AAAAAAGCGGCCGCTCACAGCCTCCTTACAGGTGACC
	TRPF3_Yeast_for	AAGCGGCCGCATGTTTTCCTCCCTGCTGCG
	TRPF3_Yeast_rev	AAAAAAGCGGCCGCTTACATCGAGCTCCGTTTAATCG
	TRPF4_Yeast_for	AAAAAAGCGGCCGCATGCCCATCCGCGTCAGGT
	TRPF4_Yeast_rev	AAAAAAGCGGCCGCGATGTGATGTATTTTATTCCCAGTTCGC
	TRPY1_Yeast_for	AAAAAAGCGGCCGCATGGTATCAGCCAACGGCG
	TRPY1_Yeast_rev	AAAAAAGCGGCCGCTTACTCTTTCTTATCCTTTATGTCTAATTTTTC

Underlined regions in the oligonucleotides for subcellular localization indicate restriction enzyme sites for *Pac*I and *Fse*I.

Italicized CC in reverse oligonucleotides for subcellular localization indicate a part of a five amino acid linker between the *TRPF* genes and the fluorescent tag not covered by the *Fse*I site.

Underlined regions in the oligonucleotides for the generation of deletion cassettes indicate universal overhangs for fusion PCR.

Italicized region in the dual purpose oligonucleotide TRPF4_5'Flank/Loc_f indicates the part needed for subcellular localization only.

Underlined regions in the oligonucleotides for Yeast complementation indicate restriction enzyme sites for *Not*I.

References:

* Krijger J-J, Horbach R, Behr M, Schweizer P, Deising HB, Wirsel SGR. The yeast signal sequence trap identifies secreted proteins of the hemibiotrophic corn pathogen *Colletotrichum graminicola*. Mol. Plant Micr. Interact. 2008; 21: 1325-1336.

\# Abou Ammar G, Tryono R, Döll K, Karlovsky P, Deising HB, Wirsel SGR. Identification of ABC transporter genes of *Fusarium graminearum* with roles in azole tolerance and/or virulence. PLOS ONE 2013; 8: e79042
