# Supplementary material for: The Transient Receptor Potential (TRP) Channel Family in Colletotrichum graminicola: A Molecular and Physiological Analysis
Source: PLoS One. 2016 Jun 30;11(6):e0158561. doi: 10.1371/journal.pone.0158561 (PMC4928787; doi:10.1371/journal.pone.0158561)
Supplement: S2 Table — Topology prediction was performed with TOPCONS (http://topcons.net/) using standard settings and the full-length protein sequences of S. cerevisiae TRPY1, and CgTRPF1, CgTRPF2, CgTRPF3, and CgTRPF4. (PDF) [file pone.0158561.s012.pdf]

S2 Table. Predicted topology of TRPY1 and CgTRPF1 through 4. Topology prediction was performed with TOPCONS (http://topcons.net/) using standard settings and the full-length protein sequences of *S. cerevisiae* TRPY1, and *C. graminicola* TRPF1, TRPF2, TRPF3, and TRPF4.

	amino acid position					element Size				
	TRPY1	TRPF1	TRPF2	TRPF3	TRPF4	TRPY1	TRPF1	TRPF2	TRPF3	TRPF4
**cytosol**	**001 - 234**	**001 - 241**	**001 - 280**	**001 - 336**	**001 - 285**	**234**	**241**	**280**	**336**	**285**
**TM1**	**235 - 255**	**242 - 262**	**281 - 301**	**337 - 357**	**286 - 306**	**21**	**21**	**21**	**21**	**21**
**vesicular**	**256 - 265**	**263 - 269**	**302 - 309**	**358 - 364**	**307 - 311**	**10**	**7**	**8**	**7**	**5**
**TM2**	**266 - 286**	**270 - 290**	**310 - 330**	**365 - 385**	**312 - 332**	**21**	**21**	**21**	**21**	**21**
**cytosol**	**287 - 298**	**291 - 303**	**331 - 342**	**386 - 397**	**333 - 349**	**12**	**13**	**12**	**12**	**17**
**TM3**	**299 - 319**	**304 - 324**	**343 - 363**	**398 - 418**	**350 - 370**	**21**	**21**	**21**	**21**	**21**
**vesicular**	**320 - 335**	**325 - 341**	**364 - 376**	**419 - 431**	**371 - 380**	**16**	**17**	**13**	**13**	**10**
**TM4**	**336 - 356**	**342 - 362**	**377 - 397**	**432 - 452**	**381 - 401**	**21**	**21**	**21**	**21**	**21**
**cytosol**	**357 - 375**	**363 - 380**	**398 - 412**	**453 - 466**	**402 - 419**	**19**	**18**	**15**	**14**	**18**
**TM5**	**376 - 396**	**381 - 401**	**413 - 433**	**467 - 487**	**420 - 440**	**21**	**21**	**21**	**21**	**21**
**putative pore region**	**397 - 436**	**402 - 442**	**434 - 484**	**488 - 529**	**441 - 479**	**40**	**41**	**51**	**42**	**39**
**TM6**	**437 - 457**	**443 - 463**	**485 - 505**	**530 - 550**	**480 - 500**	**21**	**21**	**21**	**21**	**21**
**cytosol**	**458 - 489**	**464 - 526**	**506 - 535**	**551 - 612**	**501 - 534**	**32**	**63**	**30**	**62**	**34**
TM7	490 - 510	527 - 547	536 - 556	613 - 633	535 - 555	21	21	21	21	21
vesicular	511 - 519	548 - 689	557 - 567	634 - 1163	556 - 565	9	142	11	530	10
TM8	520 - 540	not exsistent	568 - 588	not exsistent	566 - 586	21		21		21
cytosol	541 - 675	not exsistent	589 - 707	not exsistent	587 - 689	135		119		103
