# Supplementary material for: The Transient Receptor Potential (TRP) Channel Family in Colletotrichum graminicola: A Molecular and Physiological Analysis
Source: PLoS One. 2016 Jun 30;11(6):e0158561. doi: 10.1371/journal.pone.0158561 (PMC4928787; doi:10.1371/journal.pone.0158561)
Supplement: S3 Table — Organisms were ordered by phylum and class. The sequenced strain and the NCBI Taxid of each species are given in the indicated columns. To link the proteins of this table with the tree, see column TRPY1 Homologue No. For the distinct identification of the proteins the locus tag may be used. E-values were calculated on NCBI. (PDF) [file pone.0158561.s013.pdf]

S3 Table. TRPF protein sequences used to generate the phylogenetic tree. Organisms were ordered by *phylum* and *class*. The sequenced *strain* and the *NCBI Taxid* of each species are given in the indicated columns. To link the proteins of this table with the tree, see column *TRPY1 Homologue No*. For the distinct identification of the proteins the *Locus Tag* may be used. *E-values* were calculated on NCBI.

Phylum	Class	Species	Strain	NCBI Taxid	TRPY1 Homo-loge No.	E-value	Locus tag	Source
Ascomycota	Dothideomycetes	*Mycosphaerella graminicola*	IPO323	336722	1	2e-141	MYCGRDRAFT_76604	NCBI
					2	2e-15	MYCGRDRAFT_20435	
					4	4e-12	MYCGRDRAFT_75841	
		Pyrenophora tritici-repentis	Pt-1C-BFP race 1	426418	1	4e-134	PTRG_05238.1	Broad institute [1]
					2	9e-12	PTRG_06414.1	
					3	9e-16	PTRG_08905.1	
	Eurotiomycetes	*Aspergillus fumigatus*	Af293	330879	1	1e-131	Afu3g13490	NCBI
					3	8e-19	Afu6g11300	
		Penicillium chrysogenum	Wisconsin 54-1255	500485	1	6e-122	Pc13g15030	NCBI
					3	3e-24	Pc21g21930	
	Leotiomycetes	*Botrytis cinerea*	B05.10	40559	1	1e-137	BC1T_03504	Broad institute [1]
					3	1e-15	BC1T_03018	
	Pezizomycetes	*Tuber melanosporum*	Mel28	656061	1	1e-138	GSTUM_00011244001	NCBI
	Saccharomycetes	*Candida albicans*	SC5314	237561	1 TRPY3 [2]	0	CaO19.2209	NCBI
		Debaryomyces hansenii	CBS767	284592	1	0	DEHA2C08228g	NCBI
		Lodderomyces elongisporus	NRRL YB-4239	379508	1	0	LELG_01988	NCBI
		Saccharomyces cerevisiae	S288c	559292	1 TRPY1 [3]	0	YOR087W	NCBI
		Zygosaccharomyces rouxii	CBS 732	559307	1	0	ZYRO0C15510g	NCBI
	Sordariomycetes	*Colletotrichum graminicola*	M1.001	31870	1 TRPF1 [4]	3e-138	GLRG_09114.1	NCBI
					2 TRPF2 [4]	2e-17	GLRG_10771.1	
					3 TRPF3 [4]	6e-18	GLRG_08368.1	
					4 TRPF4 [4]	9e-14	GLRG_09848.1	
		Colletotrichum higginsianum	IMI 349063	80884	1	6e-135	CH063_11552.1	NCBI
					2	3e-10	CH063_03277.1	
					3	1e-18	CH063_02273.1	
					4	1e-15	CH063_07357.1	
		Fusarium graminearum	PH-1	5518	1 TRPGz [5]	1e-123	FGSG_04178.3	NCBI
					4	2e-15	FGSG_05259.3	
		Magnaporthe oryzae	70-15	242507	1 [6]	2e-137	MGG_09828.6	NCBI
					3	4e-16	MGG_01538.6	
					4	1e-21	MGG_06118.6	
		Neurospora crassa OR74A	OR74A	367110	1	4e-106	NCU16725.7	Broad institute [1]
					2	2e-16	NCU04465.7	
					3	5e-13	NCU08283.7	
					4	2e-16	NCU06601.7	
Basidiomycota	Agaricomycetes	*Laccaria bicolor*	S238N-H82	486041	1	1e-53	LACBIDRAFT_311591	NCBI
					2a	5e-11	LACBIDRAFT_305275	
					2b	5e-08	LACBIDRAFT_297283	
	Pucciniomycetes	*Puccinia triticina*	BBBD Race 1	630390	2	1e-9	PTTG_08032T0	Broad institute [1]
	Tremellomycetes	*Cryptococcus neoformans*	grubii H99	235443	1	2e-54	CNAG_07368.2	NCBI
					2	8e-07	CNAG_03844.2	
	Ustilaginomycetes	*Ustilago maydis*	521	237631	1	4e-67	UM03685.1	NCBI
					2	1e-12	UM04857.1	

[1] In the cases where no appropriate NCBI database entry was available, *E-values* were calculated locally using appropriate databases downloaded from *Broad Institute* as indicated in column *Source*.

[2] Zhou XL, Loukin SH, Coria R, Kung C, Saimi Y. Heterologously expressed fungal transient receptor potential channels retain mechanosensitivity in vitro and osmotic response *in vivo*. Eur. Biophys. J. 2005; 34: 413-422.

[3] Palmer CP, Zhou XL, Lin J, Loukin SH, Kung C, et al. A TRP homolog in *Saccharomyces cerevisiae* forms an intracellular Ca^{2+}-permeable channel in the yeast vacuolar membrane. Proc. Natl. Acad. Sci. USA 2001; 98: 7801-7805.

[4] this study

[5] Ihara M, Hamamoto S, Miyanoiri Y, Takeda M, Kainosho M, et al. Molecular bases of multimodal regulation of a fungal transient receptor potential (TRP) channel. J. Biol. Chem. 2013; 288: 15303-15317.

[6] Nguyen QB, Kadotani N, Kasahara S, Tosa Y, Mayama S, et al. Systematic functional analysis of calcium-signalling proteins in the genome of the rice-blast fungus, *Magnaporthe oryzae*, using a high-throughput RNA-silencing system. Mol. Microbiol. 2008; 68: 1348-1365.
